# Astaxanthin-Producing Green Microalga *Haematococcus pluvialis*: From Single Cell to High Value Commercial Products

**DOI:** 10.3389/fpls.2016.00531

**Published:** 2016-04-28

**Authors:** Md. Mahfuzur R. Shah, Yuanmei Liang, Jay J. Cheng, Maurycy Daroch

**Affiliations:** ^1^School of Environment and Energy, Peking University, Shenzhen Graduate SchoolShenzhen, China; ^2^Department of Biological and Agricultural Engineering, North Carolina State UniversityRaleigh, NC, USA

**Keywords:** *Haematoccoccus pluvialis*, astaxanthin, nutraceuticals, algae cultivation and processing, biorefinery

## Abstract

Many species of microalgae have been used as source of nutrient rich food, feed, and health promoting compounds. Among the commercially important microalgae, *Haematococcus pluvialis* is the richest source of natural astaxanthin which is considered as “super anti-oxidant.” Natural astaxanthin produced by *H. pluvialis* has significantly greater antioxidant capacity than the synthetic one. Astaxanthin has important applications in the nutraceuticals, cosmetics, food, and aquaculture industries. It is now evident that, astaxanthin can significantly reduce free radicals and oxidative stress and help human body maintain a healthy state. With extraordinary potency and increase in demand, astaxanthin is one of the high-value microalgal products of the future.This comprehensive review summarizes the most important aspects of the biology, biochemical composition, biosynthesis, and astaxanthin accumulation in the cells of *H. pluvialis* and its wide range of applications for humans and animals. In this paper, important and recent developments ranging from cultivation, harvest and postharvest bio-processing technologies to metabolic control and genetic engineering are reviewed in detail, focusing on biomass and astaxanthin production from this biotechnologically important microalga. Simultaneously, critical bottlenecks and major challenges in commercial scale production; current and prospective global market of *H. pluvialis* derived astaxanthin are also presented in a critical manner. A new biorefinery concept for *H. pluvialis* has been also suggested to guide toward economically sustainable approach for microalgae cultivation and processing. This report could serve as a useful guide to present current status of knowledge in the field and highlight key areas for future development of *H. pluvialis* astaxanthin technology and its large scale commercial implementation.

## Introduction

“Green microalgae” comprise more than 7000 species growing in a variety of habitats. *Haematococcus pluvialis* (Chlorophyceae, Volvocales) is unicellular fresh water microalga distributed in many habitats worldwide. It is considered as the best natural source of astaxanthin and the main producing organism of this commercial product (Lorenz, 1999; Ranga Rao et al., 2010). Astaxanthin (3,3′-dihydroxy-ß-carotene-4,4′-dione) is a bright red secondary carotenoid from the same family as lycopene, lutein, and β-carotene, synthesized *de novo* by some microalgae, plants, yeast, bacteria, and present in many of our favored seafood including salmon, trout, red sea bream, shrimp, lobster, and fish eggs (Higuera-Ciapara et al., 2006; Ranga Rao et al., 2014). Astaxanthin contains two chiral centers and can exist in three different stereoizomers, (3*S*, 3′*S*); (3*R*, 3′*S*), and (3*R*, 3′*R*). The ratio of 1:2:1 of these isomers is obtained during chemical synthesis of this compound. *H. pluvialis* biosynthesizes predominantly the 3*S*, 3′*S* stereoisomer, the most valuable one (Yang et al., 2013; Al-Bulishi et al., 2015). Astaxanthin synthesis in *H. pluvialis* is directly correlated in space and time with deposition of cellular reserves in lipid droplets under conditions of cellular stress (Solovchenko, 2015). From biochemical perspective astaxanthin is synthesized through carotenoid pathway from glyceraldehyde-3-phosphate and pyruvate. Both of these compounds are products of photosynthesis and/or glycolysis depending on cultivation conditions. These two key metabolic intermediates then enter non-mevalonate (MEP) pathway to generate IPP—key intermediate for the synthesis of all carotenoids including astaxanthin. Astaxanthin has a wide range of applications in the food, feed, cosmetic, aquaculture, nutraceutical, and pharmaceutical industries because of its free radical scavenging capacity. In terms of antioxidant activity astaxanthin is 65 times more powerful than vitamin C, 54 times stronger than β-carotene, 10 times more potent than β-carotene, canthaxantin, zeaxanthin, and lutein; and 100 times more effective than α-tocopherol (Miki, 1991; Borowitzka, 2013; Koller et al., 2014; Pérez-López et al., 2014; Cyanotech, 2015). Currently, over 95% of the astaxanthin available in the market is produced synthetically; while *H. pluvialis* derived natural astaxanthin corresponds to < 1% of the commercialized quantity (Koller et al., 2014). Synthetic astaxanthin, synthesized from asta-C_15_ -triarylphosphonium salt and the C_10_ -dialdehyde in a Wittig reaction (Krause et al., 1997), has 20 times lower antioxidant capacity than its natural counterpart and to date has not been approved for human consumption (Lorenz and Cysewski, 2000; Koller et al., 2014). Moreover, there are concerns about the safety of using synthetic astaxanthin for direct human consumption due to both different stereochemistry and potential carryover of synthesis intermediates. These concerns make natural astaxanthin from *H. pluvialis* a preferred choice for high-end markets (Li et al., 2011). In addition, *H. pluvialis* has been already approved as a color additive in salmon feeds and as a dietary-supplement ingredient for human consumption in the USA, Japan, and several European countries (Yuan et al., 2011). Nevertheless, there is no EFSA (European Food Safety Authority) approval for the therapeutic application so far. Supercritical CO_2_ extracts from *H. pluvialis* have been granted “novel food” Status by the UK Food Standards Agency (FSA), whilst US FDA (Food and Drug Administration) granted astaxanthin from *H. pluvialis* “GRAS” status (Generally Recognized As Safe) (Grewe and Griehl, 2012; Capelli and Cysewski, 2013).

The increasing demand for natural astaxanthin and its high price raises interest in efficient systems to produce astaxanthin from *H. pluvialis*. Various cultivation and astaxanthin production methods in photoautotrophic, heterotrophic, and mixotrophic growth conditions, both indoors; in open raceway ponds or closed photobioreactors using batch or fed-batch approach have been reported (Kang et al., 2005, 2010; Kaewpintong et al., 2007; Ranjbar et al., 2008; García-Malea et al., 2009; Issarapayup et al., 2009; Zhang et al., 2009; Li et al., 2011; Han et al., 2013; Wang et al., 2013a,b). The most recent advances in *H. pluvialis* cultivation for astaxanthin production include a two-stage mixotrophic culture system (Park et al., 2014) and an “attached cultivation” system using the immobilized biofilm (Zhang et al., 2014). Along with the cultivation process, the induction of carotenoid synthesis in *H. pluvialis* has a direct correlation with the astaxanthin content of cells and total astaxanthin productivity. The accumulation of astaxanthin is affected by both environmental factors such as light (Saha et al., 2013; Park et al., 2014); temperature (Yoo et al., 2012); pH (Hata et al., 2001); salt concentration (Kobayashi et al., 1993); and nutritional stresses (Boussiba et al., 1999; Chekanov et al., 2014), as well as various plant hormones and their derivatives (Yu et al., 2015). Attempts were made to genetically enhance the capacity of *H. pluvialis* to produce astaxanthin using both classical mutagenesis (Hu et al., 2008), and more recently genetic engineering (Sharon-Gojman et al., 2015). Once biomass is successfully grown and achieved high cell density, efficient harvesting, cell disruption, dehydration, and recovery/extraction of astaxanthin from *H. pluvialis* biomass are important factors. Harvesting have been so far achieved through a combination of passive settling and subsequent centrifugation (Han et al., 2013; Pérez-López et al., 2014), or flotation and centrifugation (Panis, 2015). Mechanical processes such as expeller pressing and bead milling are commonly used cell disruption methods to enhance recovery of astaxanthin from *H. pluvialis* (Razon and Tan, 2011). For the dehydration of *H. pluvialis* biomass, spray drying (Li et al., 2011; Panis, 2015), freeze drying, lyophilization, and cryodesiccation (Milledge, 2013) methods have been utilized. There are several methods of astaxanthin extraction utilizing solvents, acids, edible oils, enzymes, pressurized liquids (Jaime et al., 2010; Zou et al., 2013; Dong et al., 2014), supercritical carbon dioxide (SC-CO_2_) (Wang et al., 2012; Reyes et al., 2014), and liquefied dimethyl ether (DME) (Boonnoun et al., 2014). Although many studies have explored various methods of extraction and downstream processing of astaxanthin from *H. pluvialis* biomass, more comprehensive investigation is required to take an advantage of the biological potential of this microalga and its highly valuable product. Since astaxanthin has a great potential in the global market (280 mt, $447 million in 2014 for both synthetic and natural astaxanthin) and high market value ($2500–7000/kg); (Koller et al., 2014; Pérez-López et al., 2014; Industry Experts, 2015), in depth investigation of *H. pluvialis* biology, physiology, efficient culture techniques, downstream bioprocessing, and product formation are highly desired for further development of this sector. Even though currently several commercial companies (Cyanotech Corporation, Mera Pharmaceuticals Inc, AIgatechnologies, Fuji Chemical Industry Co. Ltd etc.) are involved in large scale production of *H. pluvialis* and astaxanthin, the production capacity is far beyond the global demand of natural astaxanthin.

This review summarizes both classical knowledge and most recent advances in the cell biology, physiological, and biochemical characteristics, responses to environmental stresses, and their effect on astaxanthin accumulation, genetic engineering, growth conditions, and different cultivation techniques, harvesting, and post harvest downstream bioprocessing of *H. pluvialis*. The biorefinery concept, global market potential, challenges, and future direction for development of *H. pluvialis* and astaxanthin production in commercial scale also are discussed.

## Biology of *H. pluvialis*

### Taxonomy and occurrence

The freshwater unicellular biflagellate green microalgae *H. pluvialis* Flotow belongs to the class Chlorophyceae, order Volvocales and family Haematococcaseae (Bold and Wynne, 1985; Eom et al., 2006). It is also known as *Haematococcus lacustris* or *Sphaerella lacustris*. *Haematococcus* was first described by J. Von Flotow in 1844 and later in 1899 Tracy Elliot Hazen extensively presented its biology and life cycle (Hazen, 1899; Leonardi et al., 2011). *H. pluvialis* is common in small transient freshwater bodies and widely distributed in many habitats worldwide. It occurs primarily in temporary water bodies like ephemeral rain pools, artificial pools, natural and man-made ponds, and birdbaths (Czygan, 1970; Burchardt et al., 2006). This microalga can be usually found in temperate regions around the world and has been isolated from Europe, Africa, North America, and Himachal Pradeslv India (Pringsheim, 1966; Suseela and Toppo, 2006). It has been also found across diverse environmental and climate conditions: in brackish water on the rocks on the seashore (Chekanov et al., 2014); freshwater basin in the rock filled with melted snow on Blomstrandhalvøya Island (Norway) (Klochkova et al., 2013); dried fountain near Rozhen, Blagoevgrad in Bulgaria Gacheva et al., 2015, freshwater fishpond in Bihor, Romania (Dragos et al., 2010); rooftop surface of a building of KIOST in Seoul Korea (Kim et al., 2015). It is well suited for survival under conditions of expeditious and extreme in light, temperature, and salt concentration that would be deleterious to many other microalgae, due to its ability to encyst (become enclosed by thick membrane) in a rapid manner (Proctor, 1957).

### Cellular morphology and life cycle

**Figure 1:**
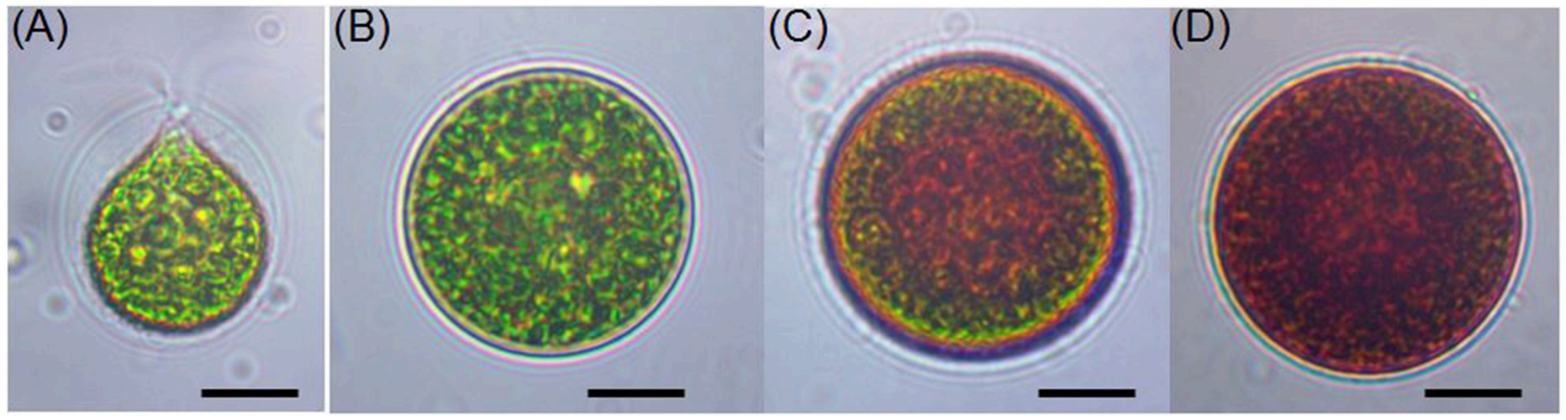
**Light microscopic images of *H. pluvialis* cells in life cycle. (A)** Green vegetative motile cell; **(B)** Green vegetative palmella cell; **(C)** Astaxanthin accumulating palmella cell in transition to aplanospore; **(D)** Astaxanthin accumulated aplanospore cell. Scale bar: 10 μm.

**Figure 2:**
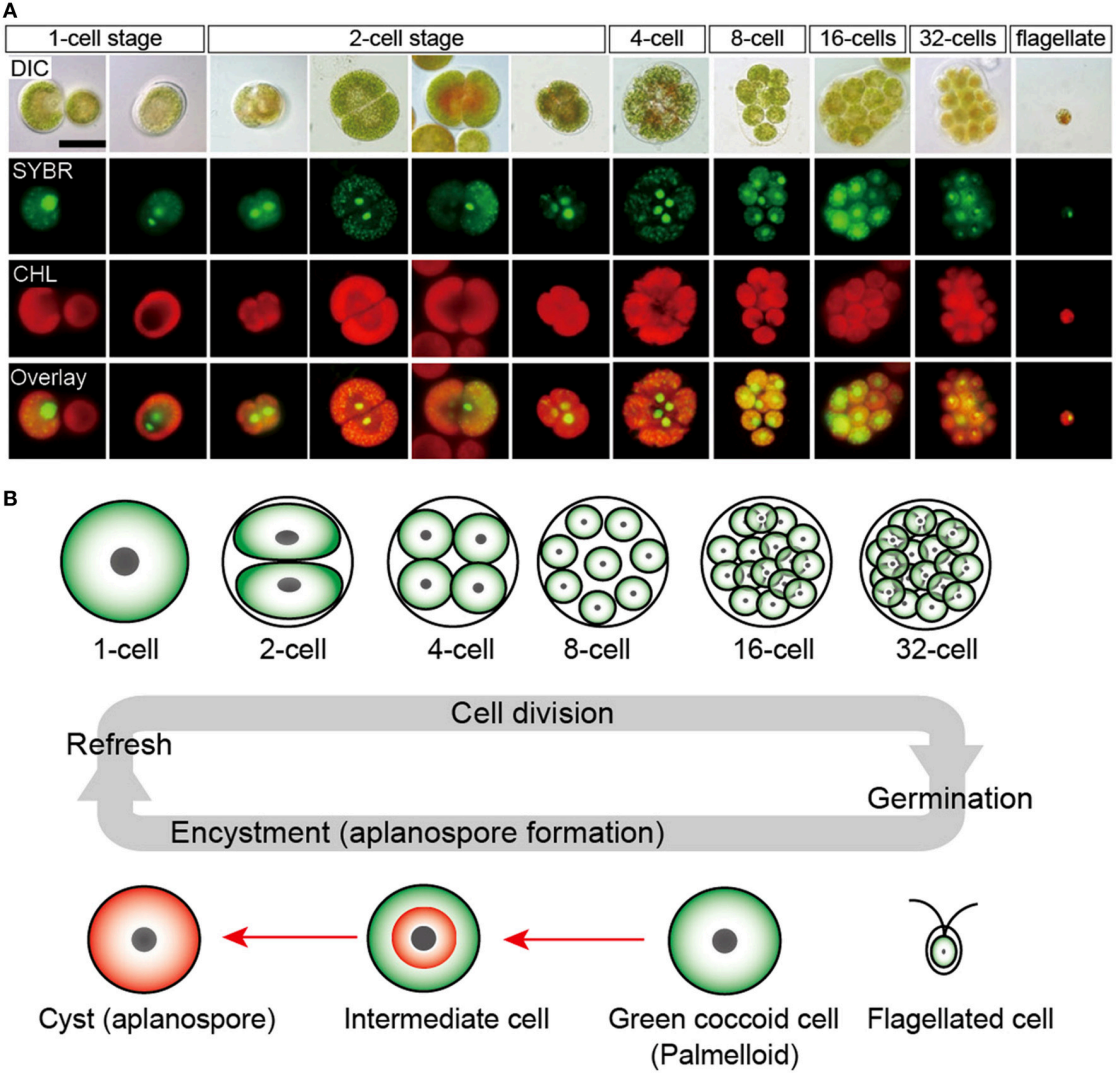
**Life cycle of *H. pluvialis*. (A)** Fluorescence microscopy images, showing the 1- to 32-cell stages, and the flagellated stage. DIC, differential interference contrast image; SYBR, SYBR Green I-stained cells (green); CHL, chlorophyll autofluorescence (red); and Overlay, overlaid images of SYBR and CHL. **(B)** Illustration of life cycle of *H. pluvialis*. Refresh, when old cultures are transplanted into fresh medium, coccoid cells undergo cell division to form flagellated cells within the mother cell wall. Germination, Flagellated cells settle and become coccoid cells. Continuous and/or strong light accelerate the accumulation of astaxanthin during encystment (red arrows). Figure reproduced from Wayama et al. (2013) distributed under the terms of the Creative Commons Attribution License.

**Figure 3:**
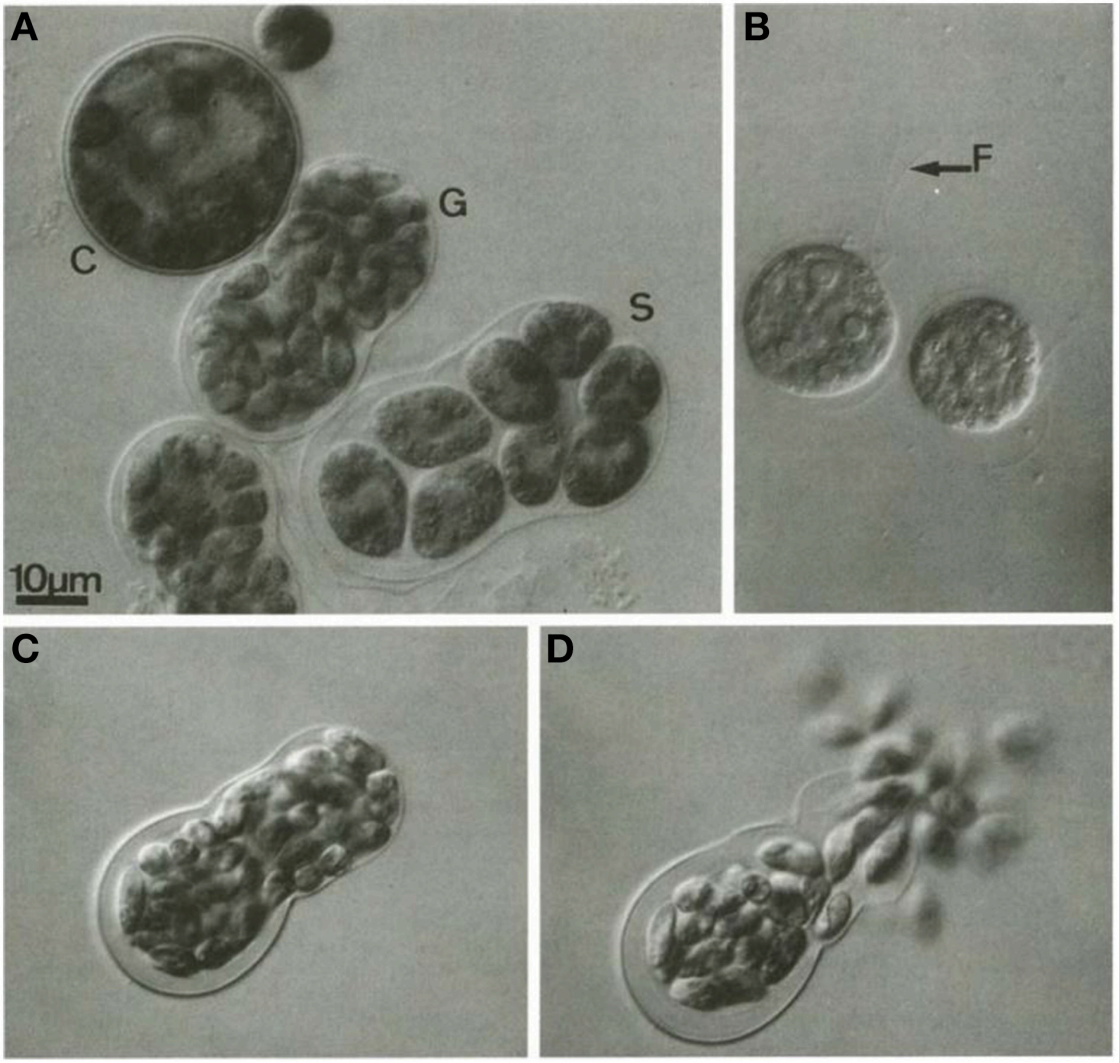
**Gametogenesis in *H. pluvialis***. **(A)** C, cyst; G, gametocyst; S, a sporocyst; **(B)** vegetative zoospore, F, flagella (indicated by arrow); **(C)**: gametocyst before releasing gametes; **(D)**: release of gametes from gametocyst. Reproduced with permission from Triki et al., 1997, Phycologia, Allen Press Publishing Services. Copyright (1997) Allen Press Publishing Services.

Cellular structure of *H. pluvialis* is similar to most of other members of volvocalean unicellular green algae. The life cycle of *H. pluvialis* consists of four types of distinguishable cellular morphologies: macrozooids (zoospores), microzooids, palmella, and hematocysts (aplanospores) (Hazen, 1899; Elliot, 1934). Macrozooids (zoospores), microzooids, and palmella stages are usually called “green vegetative phase” (Figures 1A,B). Hematocysts (aplanospores) are referred as “red nonmotile astaxanthin accumulated encysted phase” of the *H. pluvialis* life cycle (Figures 1C,D). Macrozooids (zoospores) are spherical, ellipsoidal, or pear-shaped cells with two flagella of equal length emerging from anterior end, and a cup-shaped chloroplast with numerous, scattered pyrenoids (Figure 1A). The macrozooid cells are between 8 and 20 μm long with a distinct gelatinous extracellular matrix of variable thickness. Numerous contractile vacuoles are irregularly distributed near the protoplast surface of the cell (Hagen et al., 2002). These flagellated fast-growing vegetative cells predominate under favorable culture conditions in the early vegetative growth stage (Figure 1A) Macrozooids may divide into 2–32 daughter cells by mitosis (Wayama et al., 2013) (Figures 2A,B). Under unfavorable environmental or culture conditions, macrozooids start losing flagella, and expand their cell size. They form an amorphous multilayered structure in the inner regions of the extracellular matrix or the primary cell wall as they develop into non-motile “palmella” and become resting vegetative cells (Hagen et al., 2002) (Figure 1B). With the continued environmental stress (i.e., nutrient deprivation, high light irradiance, high salinity) and cessation of cell division, “palmella” transform into the asexual “aplanospores” (Figures 1C,D). At this stage, cells contain two distinct structures, a thick and rigid trilaminar sheath, and secondary cell wall of acetolysis-resistant material. Such cells become resistant to prevailing extreme environmental conditions (Santos and Mesquita, 1984; Boussiba and Vonshak, 1991). Mature aplanospores; accumulate large amounts of secondary carotenoids, particularly astaxanthin, in lipid droplets deposited in the cytoplasm, which results in a characteristic bright red color of these cells (Hagen et al., 2002). Some *H. pluvialis* strains are reported to be capable of accumulating astaxanthin without forming aplanospores (Brinda et al., 2004). Once environmental or culture conditions return to optimal, red aplanospores germinate to form flagellated zoospores to initiate a new vegetative growth cycle (Figure 1A). In some cases, gametogenesis may occur in aplanospores (Figures 3A–D). Such process requires an exposure to extreme adverse conditions (freezing, desiccation, or nutrient starvation) followed by return to favorable culture conditions. During gametogenesis, aplanospore cells can produce up to 64 gametes which are known as microzooids. The microzooids are smaller in size (< 10 μm) compared to the zoospores (20–50 μm), and exhibit high-speed motility after their release from gametocysts. Sexual reproduction is rarely observed in *H. pluvialis*, and has been largely replaced by vegetative reproduction (Triki et al., 1997).

### Ultrastructural changes of *H. pluvialis* during the life cycle

**Figure 4:**
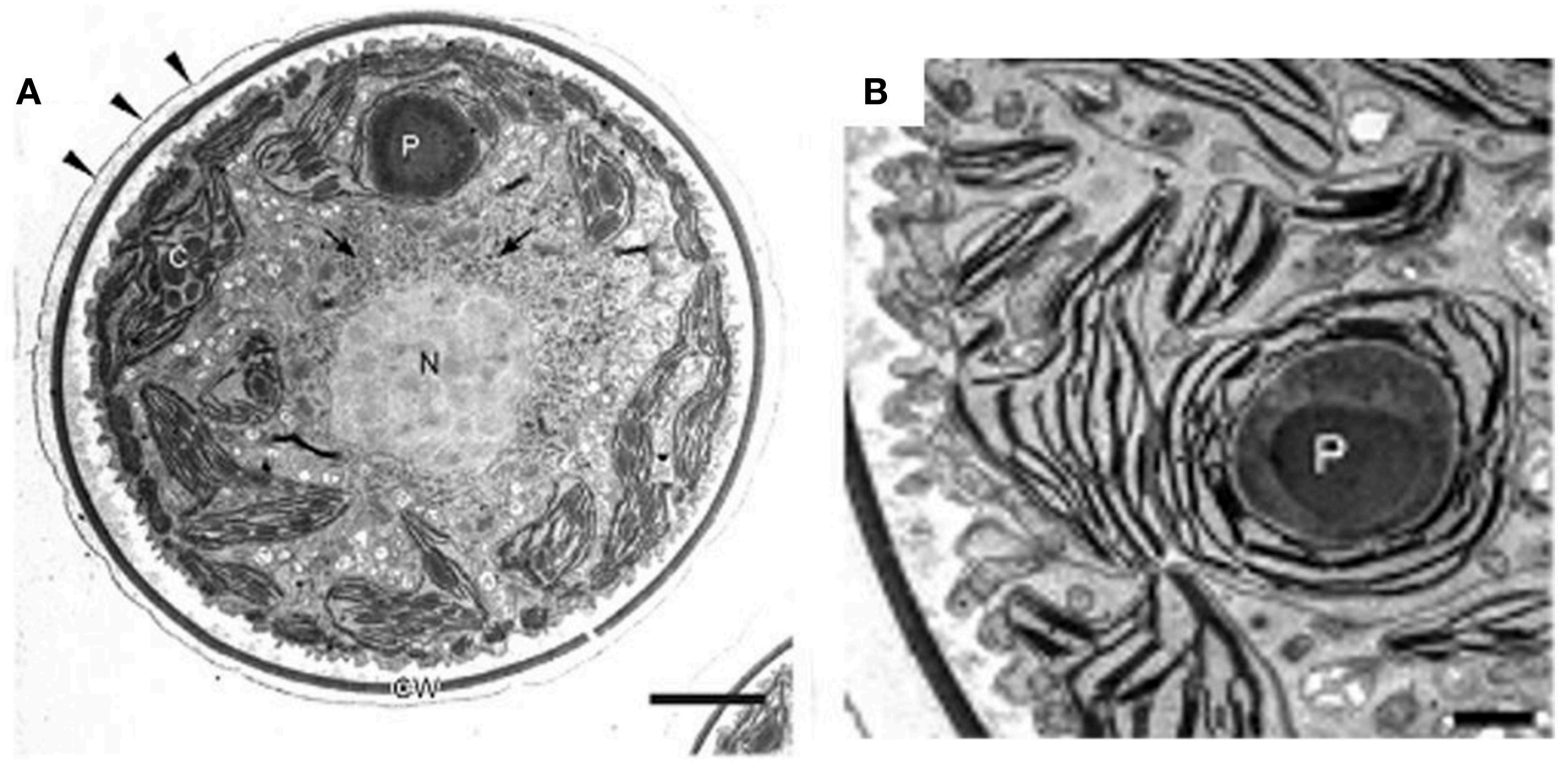
**Transmission electron micrographs of green vegetative cells of *H. pluvialis*. (A)** General ultrastructure. The cell wall is surrounded by extracellular matrix (arrowheads). Arrows indicate astaxanthin granules. **(B)** Chloroplast and pyrenoid. C, chloroplast; CW, cell wall; N, nucleus; P, pyrenoid. Scale bars in **(A,B)**: 5 and 1 μm, respectively. Figure reproduced from Wayama et al. (2013) distributed under the terms of the Creative Commons Attribution License.

**Figure 5:**
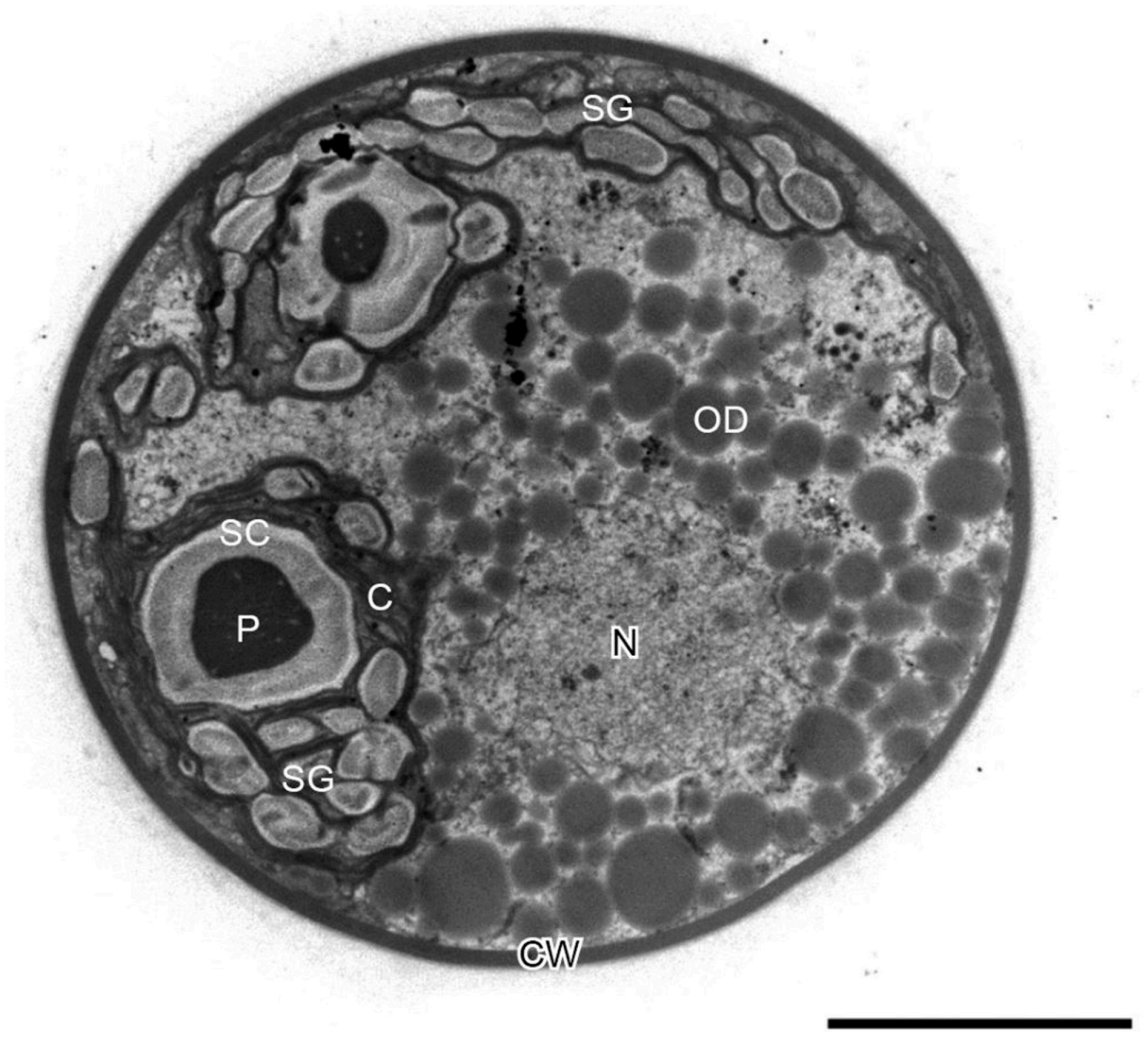
**Transmission electron micrograph of intermediate *H. pluvialis* cell (general ultrastructure)**. C, chloroplast; CW, cell wall; N, nucleus; OD; oil droplet; P, pyrenoid; SC, starch capsule; SG, starch grain. Scale bar: 5 μm. Figure reproduced from Wayama et al. (2013) distributed under the terms of the Creative Commons Attribution License.

**Figure 6:**
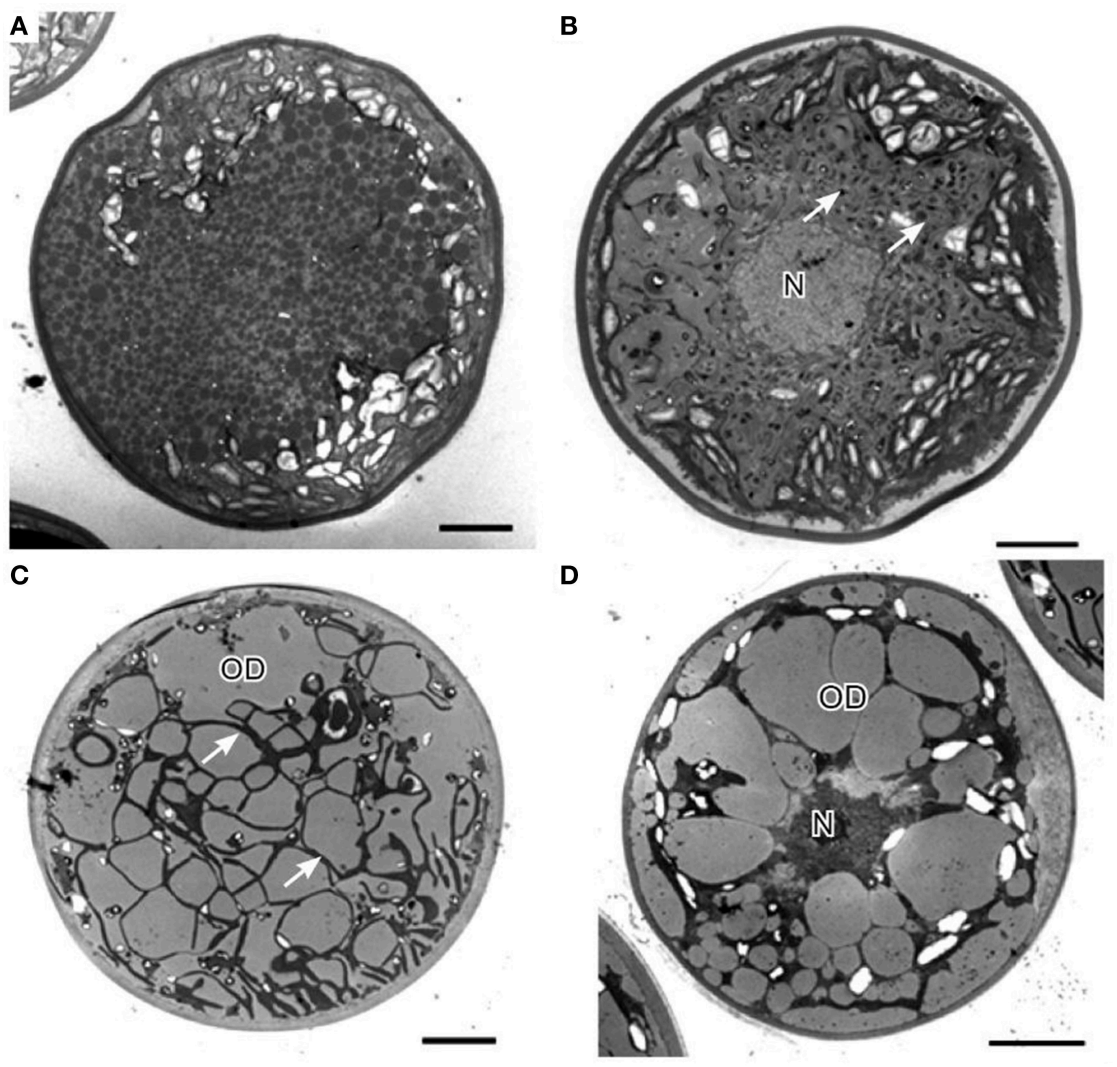
**Transmission electron micrographs of *H. pluvialis* cyst cells. (A)** General ultrastructure of cyst cells, showing small granules that contain astaxanthin. **(B)** General ultrastructure of a cyst cell, showing astaxanthin accumulation in oil droplets. **(C)** General ultrastructure of a cyst cell, showing large oil droplets. Chloroplasts localize in the interspace between oil droplets (arrows). **(D)** Some oil droplets are fused. C, chloroplast; N, nucleus; OD, oil droplet. Scale bars in **(A–D)**: 5 μm. Figure reproduced from Wayama et al. (2013) distributed under the terms of the Creative Commons Attribution License.

In *H. pluvialis* cells, large amount of ultrastructural changes occurs during their life cycle which is frequently associated with responses to stress conditions in the environment. In the green vegetative palmella cells, a thick cell wall surrounds the cell, and two layers of extracellular matrix present near the cell wall (Figure 4A). Nucleus is located in the center of the cell, and highly developed chloroplasts are located at the periphery (Figure 4A). Few astaxanthin granules are present which located around the nucleus (Figure 4A). Conspicuous pyrenoids with electron-dense matrix can be found in the stroma (Figures 4A,B) (Wayama et al., 2013). During the starting of encystment process, *Haematococcus* turned into greenish-orange cells (with some astaxanthin accumulation) which can be referred as intermediate stage cells. In this stage, conspicuous pyrenoids with electron-dense matrix located in the stroma, remain surrounded by thick starch capsules and many starch grains are located around the pyrenoids (Figure 5). Round oil droplets with various sizes, containing astaxanthin located around the nucleus (Figure 5). At this stage, thylakoids become partial degraded (Wayama et al., 2013). With the gradual accumulation of astaxanthin chloroplast reduce in volume but photosynthetic activity remains. In the aplanosopre or cyst stage, astaxanthin accumulates and cells form cysts. Oil droplets and astaxanthin accumulation patterns may differ among cyst cells. For example, electron-dense astaxanthin granules in oil droplets (Figures 6A,B) occurred in some cells. In other cells, relatively large oil droplets occurred throughout the cell (Figures 6C,D). Chloroplasts are degenerated and localized in the interspace between oil droplets (Wayama et al., 2013).

## Biochemical composition of *H. pluvialis*

Because of the unique life cycle of *H. pluvialis*, cellular composition of this microalga varies tremendously between its “green” and “red” stages of cultivation (Table 1).

**Table 1:** **Typical composition of *H. pluvialis* biomass in green and red cultivation stages**.

**Composition content (% of DW)**	**Green stage**	**Red stage**
Proteins	29–45	17–25
Lipids (% of total)	20–25	32–37
Neutral lipids	59	51.9–53.5
Phospholipids	23.7	20.6–21.1
Glycolipids	11.5	25.7–26.5
Carbohydrates	15–17	36–40
Carotenoids (% of total)	0.5	2–5
Neoxanthin	8.3	n.d
Violaxanthin	12.5	n.d
β-carotene	16.7	1.0
Lutein	56.3	0.5
Zeaxanthin	6.3	n.d
Astaxanthin (including esters)	n.d	81.2
Adonixanthin	n.d	0.4
Adonirubin	n.d	0.6
Canthaxanthin	n.d	5.1
Echinenone	n.d	0.2
Chlorophylls	1.5–2	0

Adapted from Grewe and Griehl (2012). n.d., no data.

### Protein

In green stage, during favorable growth conditions most *H. pluvialis* strains are rich in protein (29–45) (Table 1), lower protein content (23.6%) have been however observed in a Bulgarian strain *Haematococcus* cf. *pluvialis* Rozhen-12 during green stage cultivation (Gacheva et al., 2015). It was estimated that proteins contribute to 21 (Kim et al., 2015) and 23% (Lorenz, 1999) of cellular content during red stage cultivation of *H. pluvialis*. Amino acid composition of proteins in the red stage indicated that proteins were mainly composed of aspartic acid, glutamic acid, alanine, and leucine with total amino acid content of 10.02/100 mg, 46.0% of which belonged to essential amino acids (Lorenz, 1999; Kim et al., 2015).

### Carbohydrates

In green stage, carbohydrate content approximates to 15–17%, about a half of the red stage (Table 1). In the red stage, under conditions of stress (e.g., nutrient starvation, light stress, high acidity, temperature variations etc.), *H. pluvialis* accumulates higher content of carbohydrates (starch), for example 38 (Lorenz, 1999), 60 (Recht et al., 2012), and 74% (Boussiba and Vonshak, 1991). Under prolonged stress conditions starch is consumed in the cell.

### Lipid

In green stage, total lipid content varies from 20 to 25%, with approximately 10% lipids composed predominantly of short (C16, C18) polyunsaturated fatty acids deposited in the chloroplasts. Neutral lipids are predominant lipid class in both green and red cells (Table 1). In red stage, prolonged stress conditions direct larger flux toward the synthesis of neutral lipids—triacylglycerols (TAG). Red stage cells can accumulate up to 40% of their cell weight as cytoplasmic lipid droplets (LD), and considerable amount of secondary metabolites including up to 4% of the ketocarotenoid astaxanthin (Boussiba et al., 1992, 1999; Saha et al., 2013). The phospholipid content does not change compared to the green stage, while the glycolipid fraction nearly doubles in red cells when compared with green vegetative cells (Damiani et al., 2010). The total fatty acid profile of *H. pluvialis* is relatively complex. Palmitic (16:0), linoleic (18:2), and linolenic (18:3) acids are predominant components of the profile with highly polyunsaturated species also present in considerable amounts (Table 2). Based on the comparative studies on fatty acids profile of two different *H. pluvialis*, it was revealed that both strains varied in composition, especially of palmitic (16:0), oleic (18:1), linoleic (18:2), and linolenic (18:3) acids. This variation might be associated with several factors such as culture environment, stress conditions, culture parameters, variation of strain origin, nutrient etc. Higher lipid content of *H. pluvialis* grown under nutrient starvation and the suitable profile of its fatty acids indicate a possibility of biodiesel production from this microalga (Damiani et al., 2010; Saha et al., 2013). The massive astaxanthin accumulation in *H. pluvialis* is a cellular response to stress conditions and is accompanied by the enhanced biosynthesis of triacylglycerols (TAG) (Zhekisheva et al., 2002, 2005; Cerón et al., 2007), and the reduction in photosynthetic activity of PSII, loss of cytochrome *f*, and subsequent reduction in electron transport, and increased respiration rate (Boussiba, 2000). During transition from green vegetative cells to red aplanospores after exposure to stress conditions astaxanthin start to accumulate as fatty acid mono- or diesters in cytoplasmic lipid droplets (LD) (Aflalo et al., 2007). As cells undergo transition to red stage, both chlorophyll and protein contents drop.

**Table 2:** **Comparison of fatty acid composition (%) of two different *H. pluvialis* strains**.

**Fatty acids**	***Haematococcus* sp. KORDI03 (Kim et al., 2015)**	***H. pluvialis* (Lorenz, 1999)**
C12:0 lauric	N/A	0.1
C14:0 myristic	0.1	0.5
C15:0 pentadecanoic acid	0.1	N/A
C16:0 palmitic	13.7	29.0
C16:1 palmitoleic	0.5	0.6
C16:2	0.4	N/A
C16:3	3.5	N/A
C16:4	3.3	N/A
C17:0 margaric	N/A	0.2
C17:1 margaroleic	N/A	1.3
C18:0 stearic	0.7	2.1
C18:1 oleic	4.9	25.9
C18:2 linoleic	24.9	20.8
C18:3 linolenic	39.7	12.8
C18:4 octadecatetraenoic	5.8	1.4
C20:0 arachidic	N/A	0.6
C20:1 gadoleic	0.5	0.3
C20:2 eicosadenoic	N/A	1.2
C20:3 eicosatrienoic gamma	N/A	0.5
C20:4 arachidonic	0.9	1.4
C20:5 eicosapentaenoic	0.6	0.6
C22:0 behenic	N/A	0.4
C24:0 lignoceric	0.3	0.2
C24:1 nervonic acid	0.1	0.1
∑ SFAs	15.0	33.2
∑ MUFAs	6.0	28.1
∑ PUFAs	79.1	38.7
Total	100.0	100.0

### Carotenoid

The carotenoid fraction of green vegetative cells consists of mostly lutein (75–80%), β-carotene (10–20%) and others, including chlorophyll a and b, primary carotenoids, violaxanthin, neoxanthin, lactucaxanthin, and zeaxanthin (Renstrøm et al., 1981; Harker et al., 1996a). In the red stage, the total carotenoid content is markedly enhanced, and the characteristic primary carotenoid pattern of vegetative stage is replaced by secondary carotenoids, mainly astaxanthin (80–99% of total carotenoids) (Harker et al., 1996a; Dragos et al., 2010). The ratio of carotenoids to chlorophylls is about 0.2 in the green stage and increases in the red stage by an order of magnitude and reaches about 2–9. The majority of astaxanthin is not deposited in its free form but it exists within the cell as fatty acid esters of astaxanthin, usually mono- or diesters of palmitic (16:0), oleic (18:1), or linoleic (18:2) acids. This type of modification is required for the deposition of this highly polar molecule within non-polar matrix of lipid droplets. Approximately 70% monoesters, 25% diesters, and only 5% of the free ketocarotenoid is present in the mature “red” cells of *H. pluvialis* (Zhekisheva et al., 2002; Solovchenko, 2015). Under certain conditions of stress *H. pluvialis* has been shown to accumulate up to 3–5% DW of astaxanthin (Han et al., 2013; Chekanov et al., 2014).

## *H. pluvialis*-derived astaxanthin

### *H. pluvialis* as a major source of astaxanthin

*H. pluvialis* can accumulate up to 5% DW of astaxanthin and is considered as the best natural source of this high-value carotenoid pigment (Wayama et al., 2013). Dietary supplements containing *Haematococcus* astaxanthin has proved to be safe to humans and widely used for over 15 years as a nutraceutical supplement with no adverse side-effects of its supplementation (Capelli and Cysewski, 2013; Yang et al., 2013). Natural astaxanthin from *H. pluvialis* or krill oil is available in the market as a dietary supplement in dosages from 3.8 to 7.6 mg per day due to potential health benefits (Yang et al., 2013). As societies nowadays are looking toward “green” solutions, natural astaxanthin form *H. pluvialis* seems to be more favorable than its synthetic counterpart due to structure, function, application, and security (Choubert and Heinrich, 1993; Capelli and Cysewski, 2013; Pérez-López et al., 2014).

### Biosynthesis of astaxanthin in *H. pluvialis*

**Figure 7:**
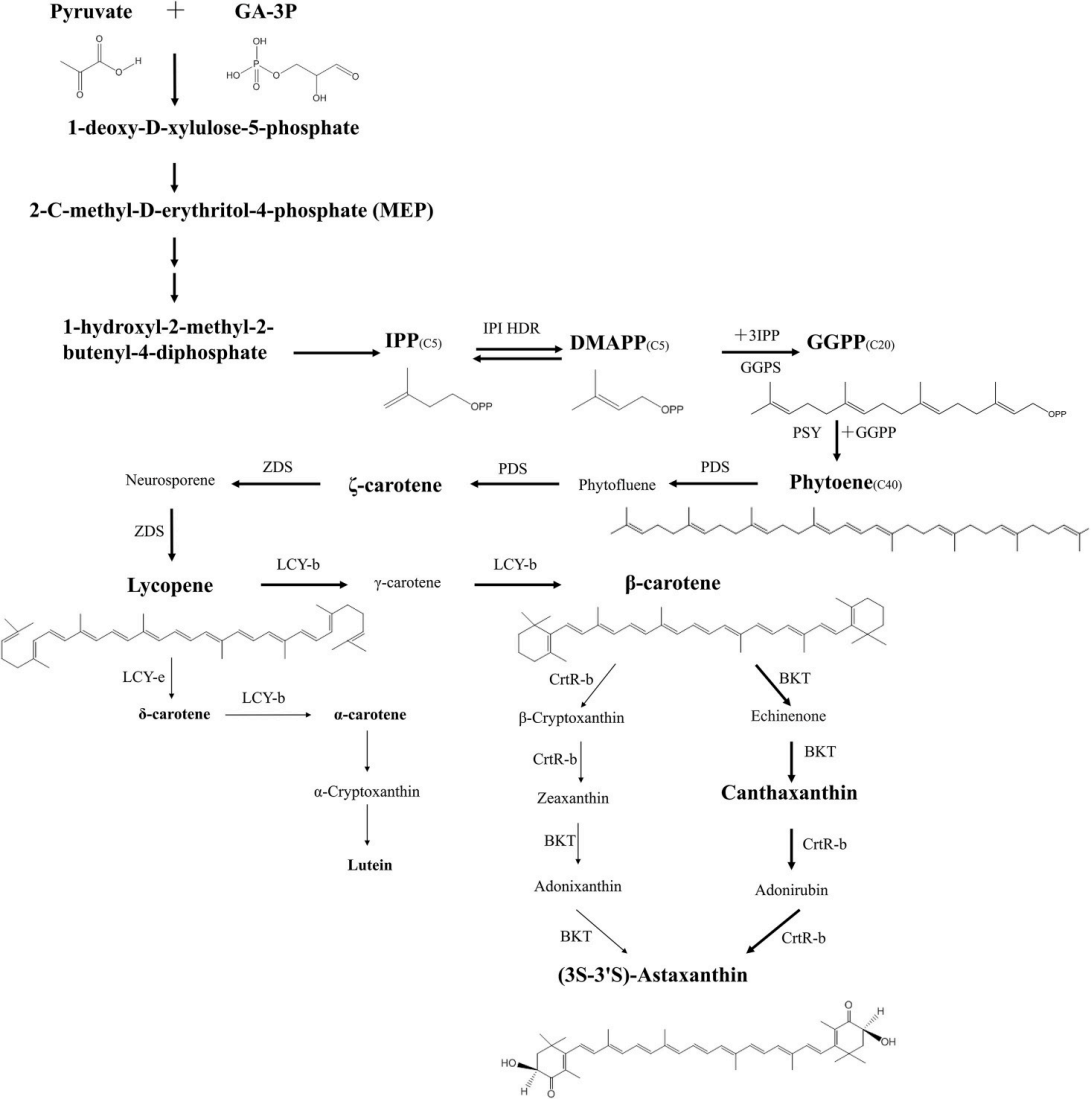
**Pathway of (3S-3′S)-astaxanthin biosynthesis in *H. pluvialis***. Major carbon flux during the red stage of *H. pluvialis* cultivation is indicated with thick arrows, minor products are indicated with thin arrows. Major intermediates of biosynthesis are indicated in large fonts, minor intermediates in small fonts. Enzyme abbreviations are as follows: IPI, Isopentenyl pyrophosphate isomerase; HDR, 4-hydroxy-3-methylbut-2-enyl diphosphate reductase; GGPS, geranylgeranyl pyrophosphate synthase; PSY, phytoenesysthase; PDS, phytoenedesaturase; ZDS, ζ-carotene desaturase; LCY-b, lycopene β-cyclase; LCY-e, lycopene ε-cyclase; BKT, β-carotene ketolase; CrtR-b, β-carotene 3,3′-hydroxylase; Intermediates: Phytofluene, Neurosporene, γ-carotene, β-Cryptoxanthin, Adonixanthin, Echinenone, Adonirubin.

Biosynthesis of astaxanthin in *H. pluvialis* is a complex process that is highly up-regulated in conditions of stress and which coincides with the accumulation of triacylglycerols (TAGs). Both compounds are deposited in the cytosolic lipid bodies during the “red” stage of *H. pluvialis* cultivation. Astaxanthin belongs to carotenoids, a C_40_ tetraterpenes, synthesized from isoprene units. Isopentenyl pyrophosphate (IPP) is a key intermediate of carotenoid synthesis. In principle, IPP can originate from two dissimilar pathways: mevalonate pathway (MVA) located in cytosol and non-mevalonate (MEP) located in the chloroplast (Lichtenthaler et al., 1997; Lichtenthaler, 1999; Eisenreich et al., 2001). Alternative name for MEP is DOXP, due to the formation of 1-deoxy-D-xylulose-5-phosphate in the first stage of the pathway. Comparative transcriptomic analysis of astaxanthin biosynthesis in *H pluvialis* have shown that the key intermediate-IPP is most likely synthesized using the DOXP pathway. *H. pluvialis* lacks three key enzymes of the mevalonate pathway (MVA) catalyzing the formation of isopentenyl pyrophosphate (IPP) from acetoacetyl-CoA (Gwak et al., 2014). There has been numerous evidence of the full set of enzymes required for the conversion of photosynthesis-derived products i.e., pyruvate and glyceraldehyde-3-phosphate into isopentenyl pyrophosphate through DOXP pathway inside *H. pluvialis* chloroplasts (Gwak et al., 2014). It makes it the most likely source of IPP in *H. pluvialis* cells. The process of astaxanthin biosynthesis is presented on Figure 7. IPP derived from DOXP pathway is an initial building block of astaxanthin synthesis. In the subsequent step the IPP undergoes isomerization to dimethylallyl diphosphate (DMAPP). It has been long assumed that this conversion was catalyzed exclusively by isopentenyl pyrophosphate isomerase (*IPI*) (Sun et al., 1998; Lichtenthaler, 1999). However, recent transcriptomic studies suggest that neither of the two *ipi* genes of *H pluvialis* (*ipi1* and ipi2*IPI2*) are up-regulated during cellular accumulation of astaxanthin (Gwak et al., 2014). Suggestions have been made that another enzyme of similar activity, 4-hydroxy-3-methylbut-2-enyl diphosphate reductase (HDR) was more likely to be responsible for catalyzing interconversion between IPP and DMAPP (Hoeffler et al., 2002; Rohdich et al., 2002; Gwak et al., 2014). Further studies are required to assess the contribution of these three enzymes to this key biosynthetic step of astaxanthin formation. Elongation of the isoprenoid chain is initiated with a molecule of DMAPP and a subsequent linear additions of three molecules of IPP catalyzed by an enzyme geranylgeranyl pyrophosphate synthase (GGPS) (Britton, 1993; Cunningham and Gantt, 1998). The final step of this process is the formation of a C_20_ compound, geranylgeranyl pyrophosphate (GGPP), a shared precursor with other isoprenoids. The first committed step of carotenoid synthesis is catalyzed by phytoene synthase (PSY) and results in a head-to-tail condensation of two GGPP molecules to form a C_40_ compound—phytoene that serves as a precursor for astaxanthin and other carotenoids (Cunningham and Gantt, 1998). The expression of the phytoene synthase gene (*psy*) was up-regulated in *Haematococcus* cells stressed with high light and undergoing transformation from “green” to “red” stage (Steinbrenner and Linden, 2001; Vidhyavathi et al., 2008; Gwak et al., 2014). The subsequent formation of highly unsaturated compound—lycopene proceeds through four desaturation steps catalyzed by two phytoene desaturases (PDS) and a ζ-carotene desaturase (ZDS) with two plastid terminal oxidase (PTOX 1, PTOX 2) acting as co-factors for electron transfer between C_40_ carotenoid intermediates, plastoquinone and final electron acceptor—oxygen (Li et al., 2010; Nawrocki et al., 2015). Of the two, PTOX 1 was found to be co-regulated with astaxanthin synthesis in *H. pluvialis* (Wang et al., 2009; Nawrocki et al., 2015). Desaturation reactions increase the number of conjugated carbon-carbon double bonds that form the chromophore in carotenoids and convert a colorless molecule of ζ-carotene to a pink colored lycopene (Cunningham and Gantt, 1998). Both termini of lycopene undergo cyclization catalyzed by lycopene cyclases (LCY-e and LCY-b). Cyclization is a branching point of the carotenoid biosynthesis in most organisms, yielding α-carotene (precursor of lutein) and β-carotene (precursor of other carotenoids including astaxanthin). In *H. pluvialis* vast majority of the carbon flux is directed into the latter (Gwak et al., 2014), and high level of LCY-b transcripts have been observed under stress conditions (Lorenz and Cysewski, 2000; Gwak et al., 2014). Final two oxygenation steps catalyzed by β-carotene ketolase (BKT) and β-carotene hydroxylase (CrtR-b) are rate limiting steps of astaxanthin synthesis (Linden, 1999; Steinbrenner and Linden, 2001; Vidhyavathi et al., 2008). Although in principle the reactions catalyzed by these two enzymes can proceed in any order, higher substrate specificity of BKT toward β-carotene than zeaxanthin favors initial addition of keto group before enantio-selective hydroxylation of canthaxanthin to astaxanthin is catalyzed by CrtR-b (Lotan and Hirschberg, 1995). Enantioselectivity of astaxanthin synthesis is of primary importance for the nutraceuticals market and the major advantage of *H. pluvialis* astaxanthin over its synthetic counterpart. Since astaxanthin has two identical chiral centers at the positions of 3 and 3′ it can exist in four different configurations which yield three different isomers: (3R, 3′S); (3R, 3′R); (3S, 3′S) depending on the spatial orientation of the hydroxyl (OH) groups in chiral carbon. During chemical synthesis these isomers are present in the ratio of 2:1:1, respectively, yielding only 25% of the naturally occurring (3S, 3′S) isoform. *H. pluvialis* synthesizes the (3S, 3′S) stereoisomer of astaxanthin and is therefore a much sought-off product in the nutraceutical market.

### Effect of small molecules on astaxanthin synthesis

Astaxanthin is a secondary metabolite, a carotenoid synthesized by *H. pluvialis* in a response to stress conditions such as high light, salinity, or carbon to nitrogen ratio (Gao et al., 2012a). Regulation of these pathways can be affected by numerous small molecules like plant hormones or their analogs. An array of such molecules has been explored to modulate astaxanthin accumulation by *H. pluvialis*. Plant hormones that are usually associated with stress response mechanisms e.g., abscisic acid (ABA), jasmonic acid (JA), methyl jasmonate (MJ) or growth regulators like gibberellic acid (GA3), salicylic acid (SA) or brassinosteroid—2, 4-epibrassinolide (EBR) were found particularly promising in increasing astaxanthin accumulation in *H. pluvialis* (Kobayashi et al., 1997b; Gao et al., 2012a,b, 2013a,b; Yu et al., 2015). It was found that these compounds affect numerous genes involved in astaxanthin biosynthesis and result in their even six to 10 fold up-regulation. All of these compounds were tested in various concentrations and the highest improvement of astaxanthin accumulation was achieved with salicylic acid. At relatively low concentrations of the hormone 50 mg L^−1^ and low light 25 μmol photons m^−2^ s^−1^ the content of astaxanthin raised seven fold from 0.391 mg L^−1^ to 2.74 mg L^−1^. Higher levels of these hormones had however deleterious effect on both growth and astaxanthin accumulation (Gao et al., 2012a). Correlation between mRNA transcript levels of five key enzymes of astaxanthin synthesis pathway (*ipi, psy, pds, crtO*, and *crtR-b* encoding respectively isopentenyl-diphosphate δ-isomerase (IPI), phytoene synthase (PSY), phytoene desaturase (PDS), β-carotene oxygenase (CrtO), and β-carotene hydroxylase (CrtR-b) with alga growth and astaxanthin production suggested a complex, multiple regulatory mechanisms at transcriptional, translational, and post-translational levels controlling entire process of carotenoid synthesis in *H. pluvialis* (Li et al., 2010). Small molecules can have multiple effects on the regulation of each of these genes and more detailed investigation of the molecular responses to their application is required for both understanding gene regulation in *H. pluvialis* and enhancing its capacity as commercial astaxanthin producer.

### Genetic improvement of *H. pluvialis* for astaxanthin production

Green eukaryotic microalgae are among the organisms that are notoriously difficult to genetically engineer. In principle, genetic engineering of microalgae has been reported for over 30 strains of various species, including *Haematococcus* (Rosenberg et al., 2008; Radakovits et al., 2010; Forján et al., 2015). In majority of these reports however, only a transient transgene expression has been achieved and a desired, stable, hereditary, and efficient genetic modification existed only for model species such as *Chlamydomonas reinhardtii* and *Volvox carteri*. Due to these constrains genetic improvement of *H. pluvialis* strains have been long limited to classical mutagenesis. Combination of mutagenic treatment and various screening methods resulted in the development of numerous interesting mutants of *H. pluvialis* including those of higher astaxanthin accumulation capacity (Tjahjono et al., 1994a; Chumpolkulwong et al., 1997; Tripathi et al., 2001; Chen et al., 2003; Hu et al., 2008; Hong et al., 2012; Gomez et al., 2013). Various mutagenic treatments have been tested, and can be broadly divided into UV and chemical mutagenesis. Chemical mutagenesis has been generally found to be more suitable for *H. pluvialis* due to alga's intrinsic capacity to limit the damage from light. Typical chemicals used for mutagenesis include ethyl methanesulphonate (EMS) and N-methyl-N-nitro-N-nitrosoguanidine (MNNG). In all studies their concentrations were adjusted to target 85–95% cell mortality. Successful mutants are usually screened using a combination of factors that promote identification of mutants capable of high astaxanthin accumulation. Typically, herbicides that affect carotenoid synthesis pathway such as norflurazon, fluridone, nicotine, compactin, or diphenylamine are used (Tjahjono et al., 1994a; Chumpolkulwong et al., 1997; Tripathi et al., 2001; Chen et al., 2003; Gomez et al., 2013). Screening relies on identifying colonies that are capable of surviving and/or growing well in the presence of inhibitory concentrations of these compounds and high illumination. Surviving strains should in principle exhibit better capacity to synthesize carotenoids and divert larger, or more efficient carbon flux toward these compounds. A number of successful mutants have been isolated this way and typical improvement of astaxanthin accumulation ranges from several percent (Gomez et al., 2013) to two or three fold improvement (Hu et al., 2008). In former case the mutated strain was tested in commercial scale cultivation system (120,000 L) and retained the improved capacity for astaxanthin production. An alternative approach to strain improvement relying on selection of photosensitive mutants was recently attempted (Hong et al., 2012). Photosensitive mutant with an ability to grow under hetero or mixotrophic conditions should be in principle advantageous over wild type strains due to faster growth rates and more efficient stress trigger. Screening for successful mutants was performed in a three stage process. Since photosensitivity is connected with impaired photosynthesis, these impaired mutants were selected in the first screening. Secondary screening tested for the ability of heterotrophic growth of these photosensitive isolates. Tertiary screening involved mixotrophic conditions with moderate illumination to obtain mixotrophic photosensitive strain that accumulated 4.7% (w/w) of astaxanthin under much shorter induction time (Hong et al., 2012). The mutated strain was stable for at least 1.5 year and is an interesting example of using classical mutagenesis for improvement of *H. pluvialis*. Mutagenic strain improvement can be expanded by breeding or creating hybrid strains from previous genetic improvement efforts. Technique of protoplast fusion has been successfully applied to *H. pluvialis* (Tjahjono et al., 1994a). Two mutagenized strains, norflurazon-resistant and nicotine-resistant have been fused to create a hybrid containing genetic material of two initial strains and showed 30% higher astaxanthin accumulation than the initial wild type strain, when neither of the fused strains showed such characteristics (Tjahjono et al., 1994a). Till very recently *H. pluvialis* was one of the organisms in which genetic engineering of its nuclear genome was considered difficult due to lack of suitable shuttle vectors and satisfactory transformation efficiencies (Sharon-Gojman et al., 2015). A number of unsuccessful approaches have been tried that included various transformation methods (particle bombardment, electroporation, *Agrobacterium*), vectors, promoters, and strains (Sharon-Gojman et al., 2015). To address these limitations and open a new array of possibilities in *H. pluvialis* and astaxanthin biology and technology new developments were required. In recent years these developments emerged and stable transformations of *H. pluvialis* chloroplast (Gutiérrez et al., 2012) and nuclear genomes (Sharon-Gojman et al., 2015) were achieved. Most recent nuclear transformation vectors are capable to transform one or two transgenes into the nuclear genome either 5′ or 3′ of the endogenous dominant selection marker, in the absence of any additional antibiotic resistance genes. The selection marker used in this system is a phytoene desaturase (*pds*) variant that confers resistance to a herbicide norflurazon due to a single point mutation (L504A). Successful transformation of *H. pluvialis* was obtained with particle bombardment and numerous constructs based on *pds* selection marker were delivered and incorporated to the genome showing stability of integration for over 16 months of subculturing (Sharon-Gojman et al., 2015). Genetic engineering of chloroplast genome of *H. pluvialis* have been also achieved relatively recently (Gutiérrez et al., 2012). So far these studies are limited to expressing exogenous antibiotic resistance gene (*aadA* cassette) between Internal Transcribed Spacer region and 16S gene of *H. pluvialis* chloroplast genome, but this technique may in the near future have significant impact on protein production in *H. pluvialis* (Gutiérrez et al., 2012) as higher protein yields are generally obtained during chloroplast expression of transgenes in other microalgal strains (Li et al., 2015). These new developments in genetic engineering of *H. pluvialis* can open a new chapter for the development of this organism as both industrial astaxanthin producer and an interesting model for carotenoid synthesis and accumulation studies.

## Applications of *H. pluvialis* astaxanthin

### Astaxanthin in human health and as nutraceutical

Astaxanthin possesses various human health benefits and nutraceutical applications and plenty of published information available with evidences, mainly from *in vitro* and animal models (Guerin et al., 2003; Chew et al., 2004; Higuera-Ciapara et al., 2006; Palozza et al., 2009; Yuan et al., 2011). The effect of *Haematococcus* derived astaxanthin on various physiological systems in animal and human subject is presented in Table 3. Astaxanthin is considered as “super anti-oxidant” which possesses one of the strongest known antioxidant effects. Its unique structure allows it to span biological membranes and act as an antioxidant by reducing and stabilizing free radicals (Hussein et al., 2006; Liu and Osawa, 2007; Ranga Rao et al., 2010). It is very good at protecting membrane phospholipids and other lipids against peroxidation (Naguib, 2000). There are several studies which showed high antioxidant activity of astaxanthin from *H. pluvialis* in rats supplemented with diet (Kamath et al., 2008; Ranga Rao et al., 2010, 2013; Augusti et al., 2012). Astaxanthin can terminate the induction of inflammation in biological systems. It can help to fight symptoms of ulcer disease from *Helicobacter pylori* (Liu and Lee, 2003); protect against gastric lesions (ulcers), improve gastrointestinal health (Nishikawa et al., 2005; Kamath et al., 2008); and treat gastrointestinal discomfort (Andersen et al., 2007; Kupcinskas et al., 2008). Astaxanthin offers protection against free radical damage to preserve immune-system defenses. The immunomodulating capacity of astaxanthin has been found to be superior to that of β-carotene and canthaxanthin (Chew and Park, 2004). Astaxanthin has shown significant effect on immune function in a number of *in vitro* and *in vivo* assays using both animal models (Chew et al., 2004) and humans (Park et al., 2010). Astaxanthin is a potential therapeutic agent against atherosclerotic cardiovascular disease (Fassett and Combes, 2011). Astaxanthin supplementation can be beneficial for people with enhanced risk for heart attacks. It is carried by VLDL, LDL, and HDL (high-density lipoprotein) in human blood and protects LDL-cholesterol against oxidation (Iwamoto et al., 2000); has a role in the reduction of blood plasma level (Karppi et al., 2007); and increases basal arterial blood flow (Miyawaki et al., 2008). Oxidative stress is a causative or at least ancillary factor in the pathogenesis of major neurodegenerative diseases (Alzheimer's, Huntington's, Parkinson's, and amyotrophic lateral sclerosis-ALS). Diets high in antioxidants offer the potential to lower the associated risks (Ferrante et al., 1997). Natural astaxanthin can cross the blood-brain barrier in mammals and can extend its antioxidant benefits beyond that barrier. Therefore, astaxanthin can help to alleviate the effects of Alzheimer's disease and other neurological diseases. Astaxanthin can improve respiratory and sympathetic nervous system activities (Nagata et al., 2006), inhibit the growth of fibrosarcoma, breast, and prostate cancer cells and embryonic fibroblasts (Palozza et al., 2009); cell death, cell proliferation and mammary tumors (Nakao et al., 2010). Astaxanthin supplementation can help to protect against UV-induced photooxidation; as an oral sun-protectant; can prevent skin thickening and reduce collagen reduction against UV induced skin damage (Ranga Rao et al., 2013) and can improve skin condition across its layers i.e., corneocyte, epidermis, basal, and dermis by combining oral supplementation and topical treatment (Seki et al., 2001; Yamashita, 2002; Tominaga et al., 2012). Results have shown that semen quality, pregnancy rate and sperm velocity in human subject can be improved (Elgarem et al., 2002; Comhaire et al., 2005) whereas idiopathic infertility can be decreased by astaxanthin (Andrisani et al., 2015).

**Table 3:** **Effect of *H. pluvialis*-derived astaxanthin on various physiological systems in human and animal subjects**.

**Physiological System**	**Subject**	**Effect followed**	**Main outcome**	**References**
Anti-oxidation	Rabbits	Thioredoxin reductase; Paraoxonase activity	Enhanced; No effect	Augusti et al., 2012
	Rats	Hepatoprotective and antioxidant activity	Improved	Ranga Rao et al., 2015
	Rats	Antioxidant enzymes, catalase, superoxide dismutase, peroxidase, and lipid peroxidation in plasma and liver	Increased	Ranga Rao et al., 2010, 2013
	Men (bilateral cataract)	Antioxidative effects through changes in superoxide scavenging activity, and hydroperoxides production in aqueous humor	Enhanced; Suppressed	Hashimoto et al., 2013
Eye function	18 healthy men	Deep vision	Improved	Sawaki et al., 2002
	10 healthy men	Eye function	Improved	Iwasaki and Tawara, 2006
	40 asthenopia patients	Eye accommodation power	Improved	Kenji et al., 2005
	49 healthy men	Uncorrected far visual acuity	Improved	Nakamura et al., 2004
	87 men (visual display terminal workers)	Eye accommodation amplitude (the adjustment in the lens of the eye that allows it to focus);	Improved;	Nagaki et al., 2006
		Eye soreness, dryness, tiredness, and blurred vision	Reduced	Nagaki et al., 2002
Skin	Healthy female or male	Skin wrinkle, corneocyte layer, epidermis, and dermis	Improved	Tominaga et al., 2012
	46 healthy women	Skin elasticity and moisture	Improved	Seki et al., 2001; Yamashita, 2002
Immune response	14 healthy women	Oxidative stress and inflammation markers; Immune response	Reduced; Improved	Park et al., 2010
Inflammation	Rats	Gastrointestinal health	Improved	Nishikawa et al., 2005
Gastric ulcer	*H. pylori*-infected mice	Bacterial load Gastric inflamation	Reduced	Liu and Lee, 2003
	Rats	Gastric ulcer markers	Reduced	Kamath et al., 2008
	44 patients with functional dyspepsia	Inflammatory markers; gastrointestinal discomfort	No effect; No effect	Andersen et al., 2007; Kupcinskas et al., 2008
Cardiovascular system	20 adult men	Blood flow time	Improved	Miyawaki et al., 2008
	Men	Blood plasma levels	Reduced	Karppi et al., 2007
Muscle endurance	16 non-trained men	Lactic acid accumulation after run	Reduced	Sawaki et al., 2002
	19 non-trained men	Respiratory and sympathetic nervous system activities	Improved	Nagata et al., 2006
	20 non-trained men	Strength/explosiveness test; strength/endurance test	No effect; Improved	Lignell, 2001
	20 resistance-trained men	Markers of skeletal muscle injury	No effect	Bloomer et al., 2005
Cancer	Rats	Growth of colon cancer cells	Inhibited	Palozza et al., 2009
Central nervous system	Healthy mice	Memory	Improved	Zhang et al., 2007
	10 healthy men (50–69 years)	Response time and accuracy of several tasks	Improved	Satoh et al., 2009
	Middle aged/elderly men and women	Cog Health battery scores (Nuropsychological memory test)	Improved	Katagiri et al., 2012
Male fertility	20 sub-fertile men	Semen quality, pregnancy rate	Improved	Elgarem et al., 2002
	30 sub-fertile men	Sperm velocity; oxidation markers; pregnancy rate	Improved; Reduced; Improved	Comhaire et al., 2005
	24 healthy men	Idiopathic infertility	Decreased	Andrisani et al., 2015
Metabolic Syndrome (MS)	Obese rats	Body weight; adipose tissue weight; MS markers	Reduced; Reduced; Improved	Ikeuchi et al., 2007

### Astaxanthin in aquaculture and poultry industry

During last 20 years, synthetic astaxanthin has been widely used for pigmentation of fish. *Haematococcus* astaxanthin has great potential in aquaculture industry, due to increasing consumer demands for natural products and ability of *Haematococcus* astaxanthin to provide necessary supplementation for adequate growth and reproduction of commercially valuable fishes (Salmonid, Red sea bream), rainbow trouts, and shrimps. Microalgae- derived astaxanthin has been demonstrated as safe and effective compound for flesh pigmentation of these fish (Torrissen and Naevdal, 1984; Tolasa et al., 2005). Utilization of *H. pluvialis* meal for pigmenting has resulted in significant astaxanthin deposition in flesh and skin, flesh coloration enhancement, enhanced antioxidant system, fish egg quality, better growth and survival of fry of Salmonid, sea bream, and rainbow trout (Arai et al., 1987; Sommer et al., 1991; Choubert and Heinrich, 1993; Sheikhzadeh et al., 2012a,b), ornamental fishes (Ako and Tamaru, 1999), and shrimp (Arai et al., 1987; Parisenti et al., 2011). A recent study indicated that diets supplemented with *H. pluvialis* can improve large yellow croaker fish growth more than diets supplemented with synthetic astaxanthin (Li et al., 2014). *H. pluvialis-*derived natural astaxanthin has shown to be efficient in pigmentation of egg yolks, egg production (Elwinger et al., 1997) in hen and breast muscle tissue improvement and higher feed efficiency in broiler chicken (Inborr and Lignell, 1997; Inbbor, 1998). It has also been proved to improve health and fertility of chicken and to decrease their mortality (Lignell and Inborr, 1999, 2000).

## Cultivation and processing of *H. pluvialis* for astaxanthin production

### Culture conditions and requirements for cell growth and astaxanthin formation

Optimization of the various culture parameters, such as growth medium composition, light, pH, temperature etc. is necessary to achieve high biomass and astaxanthin production. Most of these parameters have different optima for biomass accumulation and astaxanthin production. For carotenogenesis induction, the stronger exposure to stress conditions, the higher astaxanthin accumulation. The origins of this stress can be diverse and successful astaxanthin accumulation has been induced with both, high levels of one stressor, or from a combination of multiple stress factors. In some cases, if cells are exposed to strong stress, cells growth completely ceases and cells begin to die in a relatively short time (Su et al., 2014). Various types of growth media are used for cultivation of *H. pluvialis*. The most commonly used media are BG- 11 (Rippka et al., 1979), BBM (Bischoff and Bold, 1963), OHM (Fábregas et al., 2000); KM1-basal medium with organic carbon sources in the form of sodium acetate (Kobayashi et al., 1993), and their modifications. An ideal composition of the medium to achieve high growth rate and biomass accumulation is different from ideal composition for high accumulation of astaxanthin. Sodium nitrate was found to be the most optimal inorganic nitrogen source (Sarada et al., 2002a), alternatively an organic source such as urea can be used. When culture is subjected to nutrient deficiency, it leads to accumulation of astaxanthin within the cells (Saha et al., 2013). Nitrogen limitation leads to approximately twice the rate of astaxanthin production than the limitation of phosphorus. It can be due to higher cellular damage resulting from a lack of nitrogen, which manifests in significant degradation of chlorophyll, compared to the phosphorus starvation (Boussiba et al., 1999). Micronutrients such as selenium and chromium result in an increased biomass and astaxanthin production (Tripathi et al., 1999; Fábregas et al., 2000; Domínguez-Bocanegra et al., 2004). Formation of astaxanthin can also be induced by adding NaCl (0.25–0.5% w/v) to the media. Also, when NaCl is added together with 2.2 mM sodium acetate, astaxanthin accumulation can be increased (Sarada et al., 2002b). Addition of 0.45 mM Fe^2+^ in the form of ferrous sulfate may significantly increase the biosynthesis of carotenoids in cysts due to formation of hydroxyl radicals. (Kobayashi et al., 1997b). This effect may be enhanced by combining Fe^2+^ treatment with an addition of sodium acetate and high temperature exposure (Kobayashi et al., 1993; Tjahjono et al., 1994b). According to most studies, the suitable temperature for the growth and astaxanthin accumulation of *H. pluvialis* is between 20 and 28°C (Fan et al., 1994; Hata et al., 2001; Lababpour et al., 2005; Kang et al., 2010; Yoo et al., 2012; Wan et al., 2014a). However, temperature above 30°C induces a transition from green vegetative stage to red stage and formation of red cysts can be observed within 2 days. This transition is combined with a significant slowdown in growth, while astaxanthin accumulation is 2–3 times higher than at 20°C. The increased temperature is likely to affect the synthesis of astaxanthin through stimulation of oxygen radicals formation and their higher reactivity (Tjahjono et al., 1994b). It is preferred that the temperature change takes place gradually, allowing better acclimation to the new conditions (Hata et al., 2001). pH can also significantly affect the cell growth and synthesis of chlorophyll and carotenoids in *H. pluvialis*. In terms of biomass and astaxanthin production optimal pH is within the range of 7.00–7.85 (Hata et al., 2001; Sarada et al., 2002a). The typical irradiation for *H. pluvialis* cultivation ranges between 40 and 50 μmol photons m^−2^s^−1^ (Hata et al., 2001; Chekanov et al., 2014; Park et al., 2014). Optimal irradiation to achieve a high growth rates tend to be higher, namely 70 (Zhang et al., 2014), 80 (Saha et al., 2013), 90 (Fan et al., 1994), or even up to 177 μmol photons m^−2^s^−1^ (Domínguez-Bocanegra et al., 2004). These different optimal values may be caused by other cultivation parameters such as media composition, temperature, or the strain of *H. pluvialis*. During vegetative stage cultivation of *H. pluvialis*, the regular cycles of alternating light and dark 12:12 or 16: 8 h are often used (Saha et al., 2013; Park et al., 2014), but the higher density cultures are achieved with continuous illumination (Domínguez-Bocanegra et al., 2004). The best practice to date appears to be white or blue LED lighting (Saha et al., 2013) or the mixture of both at the ratio of 3:1 at 7000 lx (~95 μmol photons m^−2^s^−1^). These conditions promote morphologic changes from green vegetative cells to red cyst cells (Sun et al., 2015). Carotenogenesis is induced in cells upon exposure to higher light intensity than the corresponding light saturation point (LSP). However, specific optimum value of LSP differ between studies. The lowest intensities that have been reported utilized irradiation of around 100–150 μmol photons m^−2^s^−1^ (Zhang et al., 2014) followed by 240 (Saha et al., 2013), 345 (Domínguez-Bocanegra et al., 2004), and 480 μmol photons m^−2^s^−1^ (Chekanov et al., 2014). Lower optimal irradiation was found to be influenced by other stress conditions, such as deficiency of nutrients (Saha et al., 2013; Zhang et al., 2014) or elevated temperature (Tjahjono et al., 1994b). It proves that for effective induction of carotenogenesis excessive irradiation may not be necessary if other stressors are present. With the reduced requirements of light for cultivation in photobioreactors, the costs of cultivation can be minimized which is essential for astaxanthin production in an industrial scale. Regarding the type of illumination, the highest carotenoid content was obtained by using a continuous PAR lighting (Saha et al., 2013). An interesting alternative to an immediate change in the radiation intensity to induce the transition from the vegetative phase to carotenoid production is gradually increasing the level of lighting. Gradual increase of light intensity can result in gradual transformation of cells to cysts and can also contribute to better accumulation of astaxanthin, because the cells are capable to cope with increasing higher levels of stress (Park et al., 2014).

### Culture systems

Astaxanthin-producing *H. pluvialis* is capable of growing in photoautotrophic, heterotrophic, or mixotrophic growth conditions in indoors, open raceway ponds or closed photobioreactors in batch, fed batch, or continuous modes.

#### Photoautotrophic culture

Photoautotrophic culture of *H. pluvialis* is mainly carried out in open raceway ponds or closed photobioreactors. Typical photobioreactors used for its cultivation include tubular, bubble column and airlift photobioreactors. As the culture conditions for maximum growth and maximum astaxanthin content are mutually exclusive, a two-step cultivation strategy is commonly adopted for the commercial production. The first step, green vegetative growth phase (“green stage”) is to promote algal growth under favorable culture conditions (e.g., low light and nitrogen-replete) (Boussiba, 2000; Aflalo et al., 2007; Del Rio et al., 2007). When high cell density is reached, the culture enters into the second step, reddish inductive production phase (“red stage”), where algal cells are subjected to stress conditions such as high light intensity and nitrogen depletion, excess acetate addition, pH or salt stress, phosphate deficiency, or the addition of specific cell division inhibitors. These stress factors (either one or combination of more) induce the astaxanthin production in *H. pluvialis* (Fábregas et al., 2001; Torzillo et al., 2003; Orosa et al., 2005; He et al., 2007; Hu et al., 2008; Li et al., 2010; Choi et al., 2011). Therefore, carotenoid induction method has a direct correlation with both the astaxanthin content and total astaxanthin productivity. The optimal environmental and nutritional conditions for each stage are quite different (Del Rio et al., 2007). The reported biomass productivities in green stage and red stage ranged from 0.01 to 0.5 g L^−1^ d^−1^ and 0.01 to 4.8 g L^−1^ d^−1^, respectively. Astaxanthin productivity and astaxanthin content ranged from 0.44 to 21 mg L^−1^ d^−1^ and 0.8 to 4.8% of DW, respectively (Table 4). Astaxanthin can be also produced efficiently by *H. pluvialis* using a simpler “one-step strategy.” This strategy involves the administration of nitrate starvation and specific average irradiance in the culture medium, resulting in simultaneous algal cell growth and astaxanthin accumulation (Del Río et al., 2005; Del Rio et al., 2007; Del Río et al., 2008; García-Malea et al., 2009). At the laboratory scale and under continuous illumination, mean astaxanthin productivity of 20.8 mg L^−1^ d^−1^ has been reported for the one-step method (Del Río et al., 2008). The technical feasibility of this approach at a pilot scale was demonstrated in an outdoor tubular photobioreactor, which resulted in biomass and astaxanthin productivities of 0.7 g L^−1^ d^−1^ and 8 mg L^−1^ d^−1^, respectively (García-Malea et al., 2009). One-stage cultivation seems attractive, since it is less complicated than the two-stage process and the production of astaxanthin takes place in a continuous mode. It has however two serious drawbacks. First, the actual astaxanthin production is significantly lower compared to the two-stage approach. Second, this cultivation is unsuitable for outdoor setting, since it requires incessant illumination during night as well what makes the process too expensive (Aflalo et al., 2007). An “attached cultivation” approach was successfully applied in the induction of *H. pluvialis* for astaxanthin production. In this method green cells are cultured in the conventional water column and then deposited on the membrane to increase light stress surface area in the second phase of cultivation. Under the optimized conditions, biomass, and astaxanthin productivities in the attached cultivation system were 2.8 (3.7 g m^−2^ d ^−1^) and 2.4-fold (65.8 mg m^−2^ d^−1^) higher than those of the suspended bioreactor, respectively (Wan et al., 2014b). Other studies that used similar approach have reported higher astaxanthin productivities of 124 mg m^−2^ d^−1^ (Yin et al., 2015) and 164.5 mg m^−2^ d^−1^ (Zhang et al., 2014). Attached cultivation approach is superior to suspended induction in several aspects such as, lower water consumption and smaller risk of contamination. This indicates that attached induction approach can provide a promising way to boost economic benefits and considerably reduce production cost of astaxanthin from *H. pluvialis* (Zhang et al., 2014; Wan et al., 2014b). Recently, Park et al. (2014) established a two-stage “perfusion culture” system for *H. pluvialis* combining it with stepwise increase of light irradiance. Approach is based on repeated replacement of the growth medium. Cells are grown in a photobioreactor and are periodically retained in the cell settling chamber whilst growth medium is being replaced in the photobioreactor. Cells are later recycled to the bioreactor and can efficiently utilize fresh growth medium which is free of inhibitory metabolic by-products. Perfusion culture can provide high biomass productivities of 0.18 g L^−1^ d^−1^. Under stepwise increased light irradiance (150–450 μE/m^2^/s), cellular density of 12.3 g L^−1^ of have been obtained which is 3.09 and 1.67 times higher than batch and fed-batch processes, respectively whilst the productivity of astaxanthin reached 602 mg L^−1^ (Park et al., 2014).

**Table 4:** **Summary of various methods of *H. pluvialis* biomass cultivation and corresponding astaxanthin productivities**.

**PBRs type**	**Outdoor/Indoor**	**Mode**	Culture medium^*^	**Biomass productivity in Green stage (g L^−1^ d^−1^)**	**Biomass productivity in Red stage (g L^−1^ d^−1^)**	**Astaxanthin content (%, DW)**	**Astaxanthin productivity (mg L^−1^ d^−1^)**	**References**
Airlift column (30 L)	Indoor	Batch	Modified Bold's Basal medium	0.03	0.01	2.7	0.44^b^	Harker et al., 1996b
Tubular /open pond (25,000 L)	Outdoor	–	Modified Bold's Basal Medium	0.036–0.052	N/A	2.8–3.0	N/A	Olaizola, 2000
Tubular (50 L)	Indoor	Semi continuous	BG-11 medium	N/A	0.05	3.6	7.2^c^^,^ ^d^	Torzillo et al., 2003
Bubbling column (1.8)	Indoor	Batch	Basal inorganic culture medium	N/A	0.6	0.8	5.6^a^	Del Río et al., 2005
Airlift Tubular (55 L)		–	Inorganic medium free of acetate	N/A	0.41	1.1	4.4^a^	López et al., 2006
Bubbling column (0.5 L)	Indoor	Batch	BG-11 medium	0.5	0.21	4	11.5^b^	Aflalo et al., 2007
Tubular (200 L)	Outdoor	Batch	BG-11 medium	0.37	0.21	3.8	10.1^b^^,^ ^c^	Aflalo et al., 2007
Bubbling column (1.8 L)	Indoor	Batch	Basal inorganic culture medium	N/A	1.9	1.1	21^a^	Del Río et al., 2008
Bubbling column (1 L)	Indoor	Batch	Standard inorganic medium	0.36	0.14	3.6	12^b^	Ranjbar et al., 2008
Tubular (1.8 L), outdoor,	Outdoor	Continuous	Standard inorganic medium	N/A	0.7	1	8^a^	García-Malea et al., 2009
Open pond	Indoor	Batch	BG-11 medium	N/A	0.15	2.79	4.3^a^	Zhang et al., 2009
Flat type (1 L)	Indoor	Fed batch	NIES-C medium	0.33	0.44	4.8	14^b^	Kang et al., 2010
Airlift column	Indoor	Batch	*Haematococcus* medium (OHM)	N/A	0.14	N/A	3.3^a^	Choi et al., 2011
Bubbling column (6 L)	Indoor	Batch	NIES-C medium	N/A	0.047	N/A	1.4^a^	Yoo et al., 2012
Bubbling column (0.6 L)	Outdoor	Batch	BG-11 medium	N/A	0.58	2.7	17.1^b^^,^ ^d^	Wang et al., 2013a
Bubbling column (0.6 L)	Outdoor	Batch	BG-11 medium	N/A	0.30	3.8	16.0^b^^,^ ^d^	Wang et al., 2013b

a *Obtained from one-step culture process*.

b *Obtained from two-step culture process. Productivity value was calculated based on total time required by the “green stage” and “red stage” of cultivation*.

c Induction of astaxanthin was performed outdoors;

d *Obtained from a two-step process in which astaxanthin productivity was calculated based on time spent on the “red stage” only*.

* *Detail medium composition can be found in relevant references*.

#### Heterotrophic and mixotrophic culture

High light irradiance is often employed for enhancing astaxanthin formation in *H. pluvialis* cultures. However, light absorption and scattering caused by mutual shading of cells in large-scale cultures severely affects the productivity and quality of algal biomass and products. The high cost of illumination is another problem hindering the commercialization of *Haematococcus* products. To overcome this drawback, heterotrophic culture approach may be considered. Under heterotrophic growth conditions light is not needed as organic substrates serve as carbon and energy sources for growth and synthesis of secondary metabolites. Also, since lipid accumulation and astaxanthin biosynthesis are connected in space and time the effect of carbon source on lipid accumulation can have significant effect on overall productivity. It has been shown in *Haematococcus* and other microalgae lipid content and lipid profiles of microalgae are dependent on the cultivation conditions with various stress factors such as starvation or salt stress are efficient triggers of lipid accumulation, and can result in the alteration of fatty acid profiles due to cellular adjustment to particular stressor (Damiani et al., 2010; Lei et al., 2012; Saha et al., 2013; Chen et al., 2015). Various types of organic carbon sources have been used for heterotrophic cultivation and induction using acetate has been found effective for *Haematococcus* encystment and initiation of astaxanthin production (Kobayashi et al., 1991; Kakizono et al., 1992; Orosa et al., 2000; Hata et al., 2001; Kang et al., 2005). However, unlike many microalgae in which oversupply of easily accessible carbon in combination with nitrogen limitation yields diversion of the carbon flux toward lipid accumulation (Miao and Wu, 2006; Jia et al., 2014), *H. pluvialis* grows at a relatively low rate (0.22 d^−1^) and accumulates negligible amount of astaxanthin, too low to be considered for commercial scale production if single step cultivation is considered (Kobayashi et al., 1992, 1997a; Moya et al., 1997). Additionally, heterotrophic cultivation of *Haematococcus* increases the risk of bacterial or fungal contamination (Hata et al., 2001; Olguín et al., 2012). *H. pluvialis* can be also produced indoors mixotrophically employing an organic acid (e.g., acetate) or carbohydrates as an additional carbon and energy source (Kobayashi et al., 1993). Studies have shown that both growth and astaxanthin production can be enhanced under mixotrophic culture conditions. A final cell density of 0.9–2.65 g L^−1^ and a maximum astaxanthin content of 1–2% DW were obtained from mixotrophic cultures of *H. pluvialis* (Chen et al., 1997; Zhang et al., 1999; Wang et al., 2003). A sequential, heterophotric-photoautotrophic culture mode was also explored. Heterotrophic culture was used in the green stage to produce algal biomass, while astaxanthin production was induced in photoautotrophic culture conditions. The induction of astaxanthin accumulation was performed under nitrogen deprivation conditions and whilst using bicarbonate or CO_2_ as carbon sources. As a result, a very high cellular astaxanthin content of 7% (DW) was achieved, 3.4-fold higher than heterotrophic induction whilst astaxanthin productivity of 6.25 mg L^−1^ d^−1^ was obtained (Kang et al., 2005). Results indicate that photoautotrophic induction of astaxanthin production in *H. pluvialis* is more effective than heterotrophic one. Based upon the information obtained thus far, heterotrophic and mixotrophic culture modes are less cost-effective than the photoautotrophic one for *Haematococcus* mass culture.

### Microbial contamination and possible control measures

Since interest in commercial microalgae cultivation is increasing, microbial contaminants that hamper production by resulting in reduced biomass yield and quality received great attention recently. Mass culture of *H. pluvialis* is reported to be contaminated by fungal parasites and zooplanktonic predators (e.g., amoebas, ciliates, and rotifers), as well as other microalgae and cyanobacteria (Han et al., 2013). A parasitic chytrid/blastoclad fungus *Paraphysoderma sedebokerenses* is found to be responsible for reduced astaxanthin productivity and frequent culture collapses in commercial *Haematococcus* cultivation facilities (Hoffman et al., 2008; Strittmatter et al., 2015). Detection of contaminants is prerequisite for preventing and controlling of microbial contamination in mass microalgal culture. Methods of detection usually include microscopy and staining, flow cytometry, molecular based detection and monitoring. In order to cope with microalgae culture contamination, the techniques that are generally used include abiotic stresses such as ${\text{NO}}_{3}^{-}$ limitation, pH stress, temperature stress, light stress, toxic substances, and shear forces. There are some other techniques for parasite removal including salvage harvest, chemical agents (abscisic acid, copper sulfate), physical methods, biological methods (selective breeding and biological agents) (Carney and Lane, 2014). Recently, several patent applications relating to control of fungus *P. sedebokerenses* have been developed in the USA and China to protect production losses in commercial *Haematococcus* culture facilities across the world (McBride et al., 2013; Zhang et al., 2013; Carney and Sorensen, 2015).

### Harvesting

Harvesting remains one of the most challenging issues and a limiting factor for commercial algal biomass production. Harvesting of *H. pluvialis* refers to the selection of appropriate techniques to recover the “red” biomass, after the accumulation of astaxanthin in the cells and also that can facilitate cost-efficient astaxanthin extraction in the extraction phase. For the large scale harvesting of *H. pluvialis* centrifugation is the most common method and combined with other processes. Usually haematocysts are separated from the water through passive settling and subsequently concentrated with centrifugation (Lorenz and Cysewski, 2000; Olaizola, 2000; Li et al., 2011; Han et al., 2013; Pérez-López et al., 2014). Through the combination of these processes total suspended solid of 13.5% in the algal cake is achieved (Li et al., 2011). Flotation and disk-stack centrifugation have been also reported as another alternative for *H. pluvialis* harvest. Both showed more than 95% biomass recovery efficiency (Panis, 2015).

### Cell disruption

Different techniques have been developed in order to disrupt the algal cell and recover the intracellular metabolites. The most appropriate cell disruption methods to enhance recovery of astaxanthin from *H. pluvialis* at a commercial scale involve mechanical processes and more specifically expeller pressing and bead milling (Lorenz and Cysewski, 2000; Olaizola, 2003; Mercer and Armenta, 2011; Razon and Tan, 2011). During pressing (pulverization) microalgae cells are squeezed under high pressure in order to rupture the thick sporopollenin wall. Main advantage of expeller pressing is simple operation and minimization of contamination from external sources. Algal oil recovery efficiency of 75% can be achieved in a single step. Bead milling utilizes vessels filled with tiny glass, ceramic or steel beads that are agitated at high speeds. The dried biomass is fed in these vessels, where continuous exposure of biomass to the grinding media (beads) leads to cell-wall rupture, and subsequent release of intracellular compounds. This method is most effective when biomass concentration in the algal cake after harvesting is between 100 and 200 g/l (Greenwell et al., 2010). Both methods are reliable and widely applied for the *H. pluvialis* cells disruption at a commercial scale.

### Dehydration

In commercial scale astaxanthin production, dehydration (drying) ensures the quality of the pigment and leads to the formulation of the final product (Mata et al., 2010; Li et al., 2011). After algal cell walls have been disrupted, biomass must be processed rapidly within few hours to avoid spoilage. Thus, dehydration is a process applied prior to recovery of the desired metabolite, in order to extend the shelf-life of the algal biomass (Mata et al., 2010). The most known dehydration techniques that have been employed on microalgae are solar drying, spray drying, and freeze drying (Molina Grima et al., 2003; Brennan and Owende, 2010; Milledge, 2013). Spray drying has been considered as the most appropriate method to dry high-value microalgal products including *H. pluvialis* astaxanthin (Leach et al., 1998; Brennan and Owende, 2010; Li et al., 2011; Han et al., 2013; Milledge, 2013; Panis, 2015). The recovery efficiency of dry biomass (in powder) using this method exceeds 95% and in some occasions may approach 100% (Leach et al., 1998). After spray drying, the moisture content in “red” biomass is lowered to about 5% (Pérez-López et al., 2014). The main drawbacks of spray drying include high operational costs and the risk of microalgae pigments deterioration (Molina Grima et al., 2003). Freeze drying (lyophilization or cryodesiccation), involves the freezing of algal cake, the technique causes less damage than spray drying, but it is even more expensive, especially on a commercial scale (Milledge, 2013).

### Recovery of astaxanthin

Once the cell wall is disrupted and the biomass is fully dried, the recovery of the desired product is possible. Astaxanthin is a lipophilic compound and can be dissolved in solvents and oils. There is an abundance of astaxanthin extraction methods from *H. pluvialis* utilizing solvents, acids, edible oils, supercritical carbon dioxide (SC-CO_2_) as well as microwave-assisted and enzyme-assisted approaches. Among the recovery methods used solvent extraction and supercritical carbon dioxide (SC-CO_2_) extraction are considered as the most efficient, compatible, and widely used methods for astaxanthin extraction from *H. pluvialis*. The summary of various extraction methods of astaxanthin from *H. pluvialis* with recent updates is presented in Table 5 Supercritical carbon dioxide (SC- CO_2_) extraction has been widely used for industrial applications due to its many processing advantages. Due to low critical temperature of carbon dioxide, the SC- CO_2_ system can be operated at moderate temperatures, preventing the degradation of valuable substances (Machmudah et al., 2006). Several studies have reported experiments on supercritical CO_2_ extraction for the recovery of astaxanthin from *H. pluvialis.* Considering astaxanthin quality as the most important criterion, supercritical CO_2_ extraction is the most favorable option. Supercritical CO_2_ provides shorter extraction time and limits the use of toxic organic solvents. By contrast to most solvents, CO_2_ is relatively cheap, chemically inert, non-toxic, and stable (Guedes et al., 2011). Supercritical fluid extraction has also been tested with *Haematococcus*, aiming at improving the extraction efficiency. For instance, supercritical carbon dioxide (SC- CO_2_) coupled with ethanol or vegetable oil as a co-solvent can further increase the extraction efficiency of astaxanthin (80–90%) (Nobre et al., 2006; Krichnavaruk et al., 2008). There is an array of alternative approaches that can assist astaxanthin extraction from *H. pluvialis* such as solvents, acids, edible oils, enzymes, or pressurized liquids (Sarada et al., 2006; Kang and Sim, 2008; In, 2009; Jaime et al., 2010; Zou et al., 2013; Dong et al., 2014) Pressurized liquid extraction has several advantages over traditional solvent extraction. PLE requires shorter time, can be automated, uses less solvent, and retains the sample in an oxygen-free and light-free environment in contrast to traditional organic solvent extraction (Jaime et al., 2010). Recently, a simple method for the direct extraction of lipids from high moisture *H. pluvialis* microalgae was successfully achieved using liquefied dimethyl ether (Boonnoun et al., 2014).

**Table 5:** **Summary of astaxanthin extraction methods from *H. pluvialis***.

**Astaxanthin Extraction/Purification method**	**Astaxanthin yield/Extraction efficiency**	**References**
SC-CO_2_ at 20 MPa, 55°C and13% (w/w) ethanol for 120 min of extraction time.	83% recovery	Reyes et al., 2014
CO_2_ expanded ethanol (50% %w/w ethanol), 7 MPa, 45°C, 120 min of extraction time.	124.2% recovery	Reyes et al., 2014
SC- CO_2_ at at 20 MPa, 60°C, 2 ml of ethanol for 1 h of extraction time	2.45 mg/g DW	Fujii, 2012
SC- CO_2_, co-solvent 0.154–1% (v/v) ethanol, 7–34 MPa, 30–80°C, 0–100 min	74% recovery	Pan et al., 2012
SC- CO_2_, co-solvent 1.25–8.75% (v/v) ethanol, 30–50 MPa, 35–75°C, 210 min	87.4% recovery	Wang et al., 2012
SC- CO_2_, co-solvent 0–12% (v/v) vegetable oils, 30–50 MPa 50–80°C 300 min	51% recovery	Krichnavaruk et al., 2008
SC- CO_2_, co-solvent 30–50 MPa 40–80°C 60–240 min	84% recovery	Thana et al., 2008
SC- CO_2_, co-solvent, 1.67–7.5% (v/v) ethanol 20–55 MPa 40–80°C, 240 min	80% recovery	Machmudah et al., 2006
SC- CO_2_, co-solvent 0, 10% (v/v) ethanol 20–30 MPa, 40–60°C	90% recovery	Nobre et al., 2006
SC- CO_2_, co-solvent 0, 9.4% (w/w) ethanol, 30 MPa at 60°C	97% recovery	Valderrama et al., 2003
Cell germination (12 h), Ionic liquid (1-ethyl-3- methylimidazolium ethylsulfate) (24 h),	32.5 pg/cell	Praveenkumar et al., 2015
Direct extraction using liquefid dimethyl ether (DME) at 0.59 MPa and 25°C, without drying, cell disruption, or heating,	1 (mg/g cell)	Boonnoun et al., 2014
HCl:acetone (5:5), 70°C, 20 min	19.8 mg/g cell	Dong et al., 2014
Ultrasound in solvent (EtOH and EA), 16 min, 41°C, 40 kHz, 200 W, EtOH: ethyl acetate (20:1)	28 (mg/g)	Zou et al., 2013
Grinding three repetitions, pressurized hexane (10.3 Mpa)	35 (mg/g cell)	Jaime et al., 2010
Treating with enzymes (Viscozyme, Alcalase) at 50°C, 2 h	2649 ± 359 μg/g cell	In, 2009
Dodecane mixing 48 h, saponification with methanolic NaOH (0.02 M), sedimentation in darkness at 4°C, 12 h.	85% efficiency	Kang and Sim, 2008
Acid digestion, 2 N HCl, 70°C. Acetone extraction for 1 h	87% efficiency	Sarada et al., 2006
NaOH 30 min, Acetone (16 h)	7 (mg/g cell)	Mendes-Pinto et al., 2001
40% (v/v) acetone for 2 min at 80°C, followed by lyophilization or treatment with specific lytic enzymes	70% recovery	Kobayashi et al., 1997c

SC, supercritical; EA, ethyl acetate.

## Biorefinery approach for *H. pluvialis*

**Figure 8:**
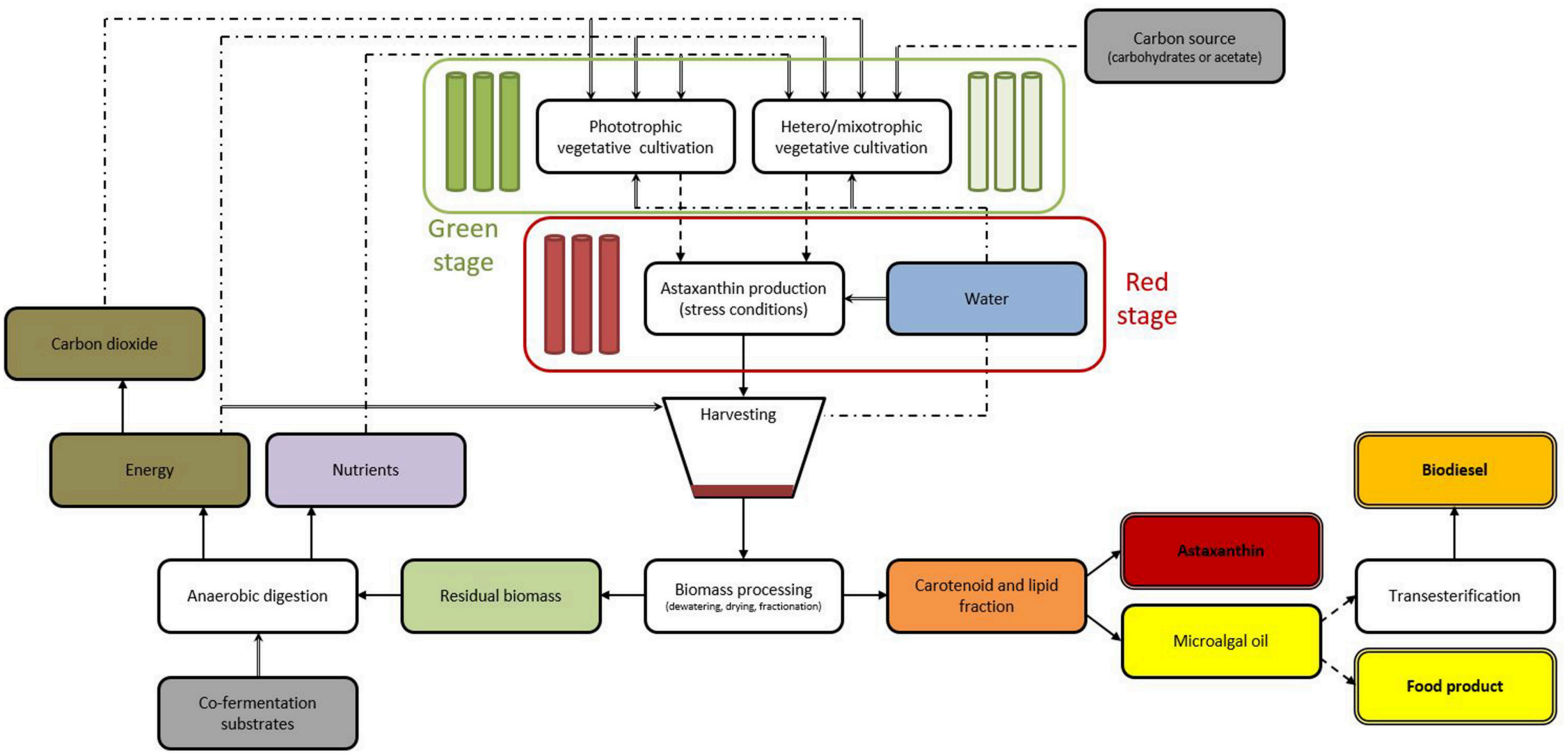
**Scheme of two stage cultivation *H. pluvialis* biorefinery producing high value compound astaxanthin and either edible oil or biofuel compound-biodiesel**. Green stage of cultivation can be performed using either photoautotrophic cultivation (deep green section) or hetero/mixotrophic cultivation (pale green section) systems. Red stage cultivation (red section) takes place after green stage of *H. pluvialis* cultivation and is aimed to maximize astaxanthin content Recycling of waste is performed through anaerobic digestion process. Following annotations are used: solid arrows—subsequent steps; dashed arrows—optional steps; double lines—final products; double arrows—inputs; dotted lines—opportunities for recycling resources.

Microalgae have been often proposed as third generation feedstock for biofuel production that does not compete for freshwater or land resources (Daroch et al., 2013a). However, despite significant advances in recent years it becomes apparent that cultivation of microalgae for the sole purpose of biofuel production is unlikely to be possible unless a major low-energy breakthrough technologies in algae cultivation, dewatering, and harvesting are developed (Li et al., 2015). In the meantime, microalgae are extremely important producers of many high-value nutraceutical compounds such as polyunsaturated fatty acids or astaxanthin that can justify high cost of microalgae cultivation and processing technologies. Integration of simultaneous production of numerous compounds within one system maximizing the benefits and limiting the costs is called biorefining (Li et al., 2015). Taking into consideration these findings *H. pluvialis* emerges as a very useful organism for the development of a dedicated microalgal biorefinery. It fits numerous requirements of for the development of first microalgal biorefineries especially the “high value product first” principle (Li et al., 2015). First, *H. pluvialis* is the best-known producer of astaxanthin-high value product worth in excess of several thousand US $ per kilogram. This product itself can easily justify expensive cultivation systems required for this organism. Second, *H. pluvialis* grown under nutrient starvation conditions induces both carotenogenesis (astaxanthin formation) and deposition of storage materials (triglycerides). It has been shown that these two responses are closely related and coincide in both space and time and triglycerides are essential for deposition of astaxanthin inside lipid bodies to confer its protective function (Solovchenko, 2015). In traditional approaches of microalgae to biofuels starvation-induced lipid accumulation is considered as significant challenge for commercialization of these systems as the overall lipid productivity of culture can drop significantly due to impaired growth rates under starvation conditions (Daroch et al., 2013b). In case of high value product like astaxanthin this drop becomes much less of the burden as the high value of the main product will compensate for this delay in final product formation. Due to the coexistence of astaxanthin and triglycerides in space and time it is possible to simultaneously obtain high value product (astaxanthin) and a biofuel feedstock (triglycerides) from a single algal feedstock. Since fatty acid content in the astaxanthin-containing ‘red’ cells can be as high as 30–60% of algae dry weight (Solovchenko, 2015) making *H. pluvialis* a very good candidate for biorefining strain. The fatty acid profiles of the alga have been evaluated by several studies and are summarized in Table 2, indicating that fatty acid profiles of the algae are suitable for biodiesel production (Damiani et al., 2010). Third, *H. pluvialis* have been found to be a mixotrophic alga what is highly advantageous for development of microalgae biorefineries. *H. pluvialis* is capable of utilizing carbon dioxide, carbonates, and carbohydrates as carbon sources, this opens a possibilities of lowering cultivation costs and/or speeding up the cultivation of the strain through using various waste streams like flue gasses or waste streams containing carbon and nutrient compounds (Wu et al., 2013). Auto-, hetero-, and mixo-trophic cultivation modes require energy and nutrients, both of which can be to an extent recycled from anaerobic digestion process. Carbon sources vary depending on cultivation mode. Photoautotrophic cultivation requires CO_2_ that can be recycled from energy production at anaerobic digestion stage. Heterotrophic cultivation requires reduced carbon source—such as carbohydrates or acetate which need to be supplied from alternative source. These compounds can also originate from waste streams. For example, food industry is rich in carbohydrate-rich waste streams that can be used in heterotrophic cultivation of *H. pluvialis* (Wang, 2014). Mixotrophic cultivation can take advantage of both sources of carbon. After simultaneous extraction of both high value product-astaxanthin and biofuel product-triglycerides algal cake composed of residual biomass can be utilized as a supplementary feedstock for biogas production using anaerobic digestion that would further assist in extraction of residual energy from this integrated bioprocess. These three features of *H. pluvialis* make it a suitable strain for the development of algal biorefineries producing high value product (astaxanthin) and biofuel molecule (biodiesel and/or biogas). The proposed biorefinery scheme is presented on Figure 8 and employs a classical two stage cultivation of *H. pluvialis* in green and red stage.

## Current global market and market players of *H. pluvialis* astaxanthin

Synthetic astaxanthin dominates current commercial market, of the total value exceeding $200 million, corresponding to 130 metric tons of product per year (Li et al., 2011). According to recent reports, microalgae-derived astaxanthin corresponds to less than 1% of the commercialized quantity due to much lower price of synthetic astaxanthin and technological problems associated with large-scale algae cultivation (Koller et al., 2014; Pérez-López et al., 2014). In recent years, there has been a growing trend toward using natural ingredients in food, nutraceutical, and cosmetic markets, resulting from increasing concerns for consumer safety and regulatory issues over the introduction of synthetic chemicals into the human food chain. The demand for natural astaxanthin derived from *H. pluvialis* in the global market has been “sky-rocketing” in recent years owing to increasing consumer awareness of its health benefits. Global market for both synthetic and natural source astaxanthin in aquaculture feed, nutraceuticals, cosmetics, and food and beverages is estimated at 280 metric tons valued at $447 million in 2014. It is further projected to reach 670 metric tons valued at $1.1 billion by 2020 (Industry Experts, 2015; Panis, 2015). Synthetic astaxanthin, astaxanthin rich *Phaffia* yeast, and *Paracoccus* bacteria are predominantly used in the aquaculture sector, while the astaxanthin derived from *H. pluvialis* is the main source for human applications such as dietary supplements, cosmetics, and food and beverages. Nowadays, the estimated market value of astaxanthin depending on products' purity varies from $2500–7000/kg to about $15,000/kg pigment from *H. pluvialis* in some cases (Borowitzka, 2013; Koller et al., 2014; Pérez-López et al., 2014; Industry Experts, 2015), while the production cost is estimated at about $1000 per kg of astaxanthin from *H. pluvialis* (Li et al., 2011). Natural astaxanthin is three to four times more valuable than the synthetic alternative in nutraceutical and pharmaceutical markets (Han et al., 2013). Since there is growing market demand for natural astaxanthin for specific commercial applications (e.g., the nutraceuticals market) to replace the synthetic astaxanthin, mass cultivation of *H. pluvialis* in industrial scale has great potential and attractive business opportunity. However, current market demand for natural astaxanthin is not met. It is expected that in the foreseeable future after the optimization of the production technology, the production costs of the natural astaxanthin from *H. pluvialis* should be more competitive to these of the synthetic alternative (Pérez-López et al., 2014). Since the mid 1990's, several leading companies are successfully producing *H. pluvialis* at commercial scale and marketing natural astaxanthin from *H. pluvialis* worldwide. The list of the leading companies with their products is presented in Table 6. The size of the nutraceutical astaxanthin market is growing day by day and this market is very attractive to *Haematococcus* astaxanthin producers since the price of these products is significantly higher than those of feed applications. *Haematococcus* producers need to invest their attention for increasing astaxanthin production capacity to meet the global demand. It is worthy to mention that several manufacturers already have doubled the cultivation capacities in recent years. Apart from an increase in existing players' capacities, new producers, such as BGG in China, have entered the market with significant production capacities.

**Table 6:** **Leading commercial companies and their *H. pluvialis-*derived astaxanthin and related products in the world market**.

**Company Name**	**Country**	**Brand Name**	**Product particulars**
Cyanotech Corporation (www.cyanotech.com)	USA	BioAstin®	Astaxanthin extract packaged in soft gel, beadlets; dietary supplement
		Naturose™	Algae meal; pigmentation source for ornamental fish and animals
Mera Pharmaceutigals Inc. (www.merapharma.com)	USA	AstaFactor®	Astaxanthin packaged as soft gel; dietary supplement
Stazen Inc. (www.stazen.com)	USA	Stazen®	Dietary supplement containing algae crushed and dried algae meal
Valensa International (www.valensa.com)	USA	Zanthin®	Astaxanthin extract, soft gel, beadlets
AIgatechnologies Ltd. (www.algatech.com)	Israel	AstaPure™	Dry algal biomass, astaxanthin beadlets, and oleoresin
Fuji Chemical Industry Co. Ltd. (AstaReal Co Tld) (www.fujichemical.co.jp; www.astareal.com)	Japan, Sweden, USA	AstaREAL®	Astaxanthin oleoresin products, water dispersible, and soluble powders
BioReaI (Sweden) AB (subsidiary of Fuji Chemical) (www.bioreal.se)	Sweden	AstaXine® AstaCaroxe®	Dietary supplement containing algae crushed, and dried algae meal
		AstaEquus®	Astaxanthin extract feed supplement for horses
		Novaasta®	Astaxanthin extract feed supplement for animals
Britannia Health Products Ltd. (www.britanniahealth.co.uk)	UK	Britaxan®	Astaxanthin complex with other carotenoids packaged as capsule- dietary supplement
Supreme Biotechnologies NZ Ltd. (www.supremebiotech.com)	New Zealand	AstaSupreme®	Algal biomass, Oleoresins, beadlets, and soft gels of Astaxanthin
Atacama Bio Natural (www.atacamabionatural.com)	Chile	Supreme Asta Oil™ Supreme Asta powder™	Oleoresin for food, nutraceutic, and cosmetic products, and powder for animal feed supplement
Jingzhou Natural Astaxanthin Inc. (www.asta.cn)	China	NaturAsta™	Dry algal biomass and astaxanthin soft gel
Kunming Biogenic Co. Ltd. (www.bgenic.com)	China	AstaBio®	Algal biomass, oleoresins, beadlets, and soft gels of Astaxanthin
Beijing Ginko Group (BGG) Biological Technology Co. Ltd. (www.gingkogroup.com.cn)	China	AstaZine®	Astaxanthin oil, powder, and beadlets
Wefirst Biotechnology Co. Ltd. (www.astawefirst.com)	China	AstaFirst™	Dried algal powder, Astaxanthin oleoresin, and soft gel
Algaetech International SDN BHD (www.algaetech.com.my)	Malaysia	Astaxanthin Premia-*Ex*	Algae biomass, oleoresin, and soft gel
Parry Nutraceuticals Ltd. (EID Parry) (www.eidparry.com/)	India	Zanthin®	Astaxanthin oleoresins, beadlets, and soft gel

## Major challenges for the improvement of *H. pluvialis* biomass and astaxanthin production

There are many challenges and problems for the development of large scale production of biomass and astaxanthin from *H. pluvialis.* Due to these obstacles the productivity can be hampered and in some cases a failure of the production system can make the production process economically unsustainable. Following issues are considered as most important challenges for the development of *H. pluvialis* astaxanthin production process:

- Lack of effective solution to prevent or treat microbial contaminations of mass cultures in a commercial scale.
- Slow cell growth rate, sensitivity of the cells to hydrodynamic stress, and changes in cell morphology under various environmental conditions.
- Inadequate and cost ineffective cultivation, drying, and astaxanthin extraction technologies at the commercial scale.
- Unavailability of genetically improved/engineered strains of *H. pluvialis* and genetic transformation tools for engineering astaxanthin biosynthesis pathways in this organism for improved astaxanthin production.
- Lack of sufficient number of skilled workers in production farms and insufficient collaboration between universities and commercial enterprises.
- Lack of adequate scientific research on the economic performance and viability of commercial scale astaxanthin production process.

## Conclusion and perspectives

This review provides an insight about the latest scientific and technological advancements in various aspects of astaxanthin-producing microalga *H. pluvialis* such as cell biology, reproduction, biosynthesis pathway, stress mechanism, biomass production, and downstream processing. It also contemplates a broader image including potential benefits, global market opportunities and integration of astaxanthin production into biorefining. In recent years there is an increased interest for natural astaxanthin from green microalga *H. pluvialis*. Wide ranges of scientific improvements have been achieved during the last decade in terms of productivity and bioprocessing in order to obtain a refined astaxanthin product. Yet its commercial production, especially for low-end markets is too expensive for mass adoption of natural astaxanthin over its synthetic counterpart. *H. pluvialis* has been shown to be cultured in photoautotrophic, heterotrophic or mixotrophic growth conditions in various culture systems. Research have been conducted on the optimization of the various culture parameters, such as growth medium composition, light, pH, temperature etc. to achieve high biomass and astaxanthin production. Most of these parameters have been optimized and found different for biomass accumulation and astaxanthin production. Little can be done to address this limitatiation as it is funadamentally connected with the life cycle of this microalgae. We believe there exist three key areas where further improvements are required and interesting novel approaches have been recently developed: cultivation efficiency and cost; good cultivation practice and predator control; and astaxanthin isolation and purification.

First, due to complex life-cycle of *H. pluvialis* it is important to maximase cell densities of alga at “green stage” of cultivation to maximize astaxanthin yield from the “red stage.” We think that a number of recent developments can make significant impact in maximizing cell densities. Especially attached cultivation approach and a two-stage “perfusion culture” system can be considered most promising due to the capability of producing several fold higher biomass and astaxanthin productivity and some other benefits such as lower water consumption and smaller risk of contamination. These improvements may boost economic benefits and reduce production cost of astaxanthin from *H. pluvialis* (Park et al., 2014; Wan et al., 2014b; Zhang et al., 2014). Alternatively, utilization of supplementary carbon source and adoption a two-stage sequential heterotrophic-photoautotrophic approach could improve biomass and astaxanthin production. Especially utilization of waste carbon and nutrient sources in biorefinery setup could help to decrease cultivation costs. Unfortunately, these researches are still in laboratory stage and need to be tested in large-scale commercial production for further validation.

Second, control of contaminants, parasites, and predators remains to be primary concern for *Haematococcus* growers and major issue in culture stability and astaxanthin productivity. Since there is very little that can be done once contamination takes place it is important to limit the possibility of such disruption and identify it as soon as possible, and avoid spreading to other parts of culture. Traditional detection methods such as microscopy and staining can be used to visualize algal parasites, however this technique may be too labor intensive to perform on a routine basis for most commercial operations. For routine detection, more automated systems such as flow cytometry would be ideal. Alternatively, molecular-based techniques that are considered as the most informative and sensitive for the detection and identification of parasites. Following techniques are worth further exploring DNA sequencing (Sanger, shotgun, or next generation) and then monitoring for these specifically using qPCR or phylochip technology. Decreasing costs of next generation DNA sequencing can make DNA sequencing for culture diagnostic purposes more accessible in the near future.

Third, combination of low cell densities and robust trilayer cell walls of astaxanthin-containing aplanospores make isolation of astaxanthin difficlut and expensive. Currentlly harvesting by centrifugation, cell wall disruption by expeller pressing and bead milling are the most common described methods for commercial scale astaxanthin production from *H. pluvialis.* After cell walls disruption, biomass is usually processed by spray drying or freeze drying. A number of astaxanthin extraction methods such as (solvents, acids, edible oils, supercritical carbon dioxide, microwave-assisted, and enzyme-assisted approaches have been reported for *H. pluvialis* and supercritical carbon dioxide (SC- CO_2_) extraction has been widely used for industrial applications. Two recently developed methods allow efficient extraction of astaxanthin-containing lipids from wet biomass at yields comparable to conventional drying-solvent extraction method. Efficient extraction of astaxanthin from wet *H. pluvialis* biomass was achieved with liquefied dimethyl ether (Boonnoun et al., 2014) and also cell germination process in conjunction with ionic liquids treatment (Praveenkumar et al., 2015).

Despite significant advances in research and development of *H. pluvialis* astaxanthin production is still in laboratory stage and often faces difficulties to become implemented in large-scale commercial production. There is a number of other areas of improvement that will contribute to the expansion of *Haematococcus* production capacity, lowering the production cost, and increasing market penetration at low end applications. These include: next-generation culture systems along with advanced management practices; better understanding of astaxanthin biosynthesis, metabolic pathways and their regulation, genetic engineering, and omics-scale understanding of astaxanthin accumulation; development of genetic manipulation toolbox; exploration of integration of *H. pluvialis* cultivation with other processes. Yet, we firmly believe that three key areas of focus should be: cultivation efficiency and cost; good cultivation practice and predator control; and astaxanthin isolation and purification. Further developments in these fields can have a profound effect on the commercial deployment of *H. pluvialis* astaxanthin products and can act as a catalyst for the development of an entire microalgae industry in the near future.

## Author contributions

MS, collected data, participated in preparation of draft manuscript, participated in assembly and editing of the final manuscript; YML, collected data, participated in preparation of draft manuscript; JJC, participated in assembly and editing of the final manuscript; MD, collected data, participated in preparation of draft manuscript, participated in assembly and editing of the final manuscript.

## Funding

Authors would like to acknowledge the support of National Natural Science Foundation of China for Young International Scientists Grant no. 31450110424 and 31550110497, Shenzhen Municipal Government for Special Innovation Fund for Shenzhen Overseas High-level Personnel KQCX20140521150255300 and Shenzhen Knowledge and Innovation Basic Research Grant JCYJ20150626110855791, State Ocean Administration Grant 201305022, and National Thousand People Plan Grant of Jay Jiayang Cheng.

### Conflict of interest statement

The authors declare that the research was conducted in the absence of any commercial or financial relationships that could be construed as a potential conflict of interest.
